# A novel homeostatic loop of sorcin drives paclitaxel-resistance and malignant progression via Smad4/ZEB1/miR-142-5p in human ovarian cancer

**DOI:** 10.1038/s41388-021-01891-6

**Published:** 2021-06-23

**Authors:** Jinguo Zhang, Wencai Guan, Xiaolin Xu, Fanchen Wang, Xin Li, Guoxiong Xu

**Affiliations:** 1 Research Center for Clinical Medicine, Jinshan Hospital, Fudan University, Shanghai, 201508 China; 2 Department of Oncology, Shanghai Medical College, Fudan University, Shanghai, 200032 China; 3 Center for Tumor Diagnosis and Therapy, Jinshan Hospital, Fudan University, Shanghai, 201508 China

**Keywords:** Ovarian cancer, Predictive markers

## Abstract

The primary chemotherapy of ovarian cancer (OC) often acquires chemoresistance. Sorcin (SRI), a soluble resistance-related calcium-binding protein, has been reported to be an oncogenic protein in cancer. However, the molecular mechanisms of SRI regulation and the role and aberrant expression of SRI in chemoresistant OC remain unclear. Here, we identified SRI as a key driver of paclitaxel (PTX)-resistance and explored its regulatory mechanism. Using transcriptome profiles, qRT-PCR, proteomics, Western blot, immunohistochemistry, and bioinformatics analyses, we found that SRI was overexpressed in PTX-resistant OC cells and the overexpression of SRI was related to the poor prognosis of patients. SRI was a key molecule required for growth, migration, and PTX-resistance in vitro and in vivo and was involved in epithelial–mesenchymal transition (EMT) and stemness. Mechanistic studies showed that miR-142-5p directly bound to the 3ʹ-UTR of SRI to suppress its expression, whereas a transcription factor zinc-finger E-box binding homeobox 1 (ZEB1) inhibited the transcription of miR-142-5p by directly binding to the E-box fragment in the miR-142 promoter region. Furthermore, ZEB1 was negatively regulated by SRI which physically interacted with Smad4 to block its translocation from the cytosol to the nucleus. Taken together, our findings unveil a novel homeostatic loop of SRI that drives the PTX-resistance and malignant progression via Smad4/ZEB1/miR-142-5p in human OC. Targeting this SRI/Smad4/ZEB1/miR-142-5p loop may reverse the PTX-resistance.

## Introduction

Ovarian cancer (OC) is ranked the most leading cause of gynecological cancer death worldwide [1]. Over the last 3 decades, the long-term survival rate has undergone little improvement in patients with advanced-stage OC. The 5-year cause-specific survival rate is about 20–41% [2]. The recommended treatment for patients with advanced OC is debulking surgery followed by platinum/taxane-based chemotherapy [3]. The majority of the patients have an initial response to chemotherapy, e.g., cisplatin and paclitaxel (PTX). A randomized phase III trial showed that compared with intravenous PTX plus cisplatin, intravenous PTX plus intraperitoneal cisplatin-PTX improves survival in patients with optimally debulked stage III OC [4] and weekly PTX is highly active [5]. However, a substantial proportion of patients eventually develop chemoresistance and relapse [6, 7], which represents the main obstacle to the treatment of advanced OC. Therefore, a better understanding of the mechanisms and exploring the clinically applicable biomarkers of chemoresistance are required to retrieve the chemosensitivity in OC patients. To define such chemo-biomarkers, we cloned PTX-resistant cells and performed microarrays (Accession #GSE168927; https://www.ncbi.nlm.nih.gov/).

One candidate of chemo-biomarkers is sorcin (SRI), an oncogenic protein in various cancers [8–11], which is involved in chemoresistance by binding to chemo-drugs with different affinity [12]. SRI is a 22-kDa soluble resistance-related calcium-binding protein that belongs to the penta-EF hand (PEF) family containing multiple E-F hand domains [13]. The *SRI* gene is located in chromosome 7q21 where a well-known multi-drug resistant gene 1 (*MDR1*, its protein name known as ATP binding cassette B1, ABCB1, P-glycoprotein, P-gp) is lodged [14] and is found to maintain the calcium homeostasis in cells, to inhibit endoplasmic reticulum stress, and to regulate vesicle trafficking [15, 16]. However, the role and molecular mechanisms of SRI in PTX-resistant OC remain unclear. miRNA (miR) is one of the factors in OC chemoresistance [17].

The aberrant expression and dysfunction of miRNA are often found in patients with chemoresistance [18]. Our preliminary analysis predicts that SRI is a target of miR-142-5p, whereas miR-142-5p has been reported to be acting as an oncogenic miRNA to promote the progression of breast cancer and colorectal cancer [19, 20]. Nevertheless, the functional role and the mechanisms of miR-142-5p in PTX-resistant OC have not been investigated and may be mediated by multiple surrounding factors. Evidence has shown that epithelial-mesenchymal transition (EMT) contributes to invasion, chemoresistance, and stem-like phenotype in various types of cancer [21]. Zinc finger E-box binding homeobox 1 (ZEB1), a core EMT-transcription factor, endows cancer cells with a mesenchymal-like and stem-like phenotype and correlates with poor clinical outcomes in human cancers [22]. ZEB1 is a transcriptional factor that regulates target genes including miRNA [23]. However, studies concerning ZEB1-related chemoresistance in OC are limited. Here, we identified and explored for the first time that SRI acts as a key driver that controls malignant progression and PTX-resistance via a Smad4/ZEB1/miR-142-5p homeostatic loop in OC.

## Results

### Sorcin is overexpressed in PTX-resistant OC related to the poor prognosis

**Fig. 1:**
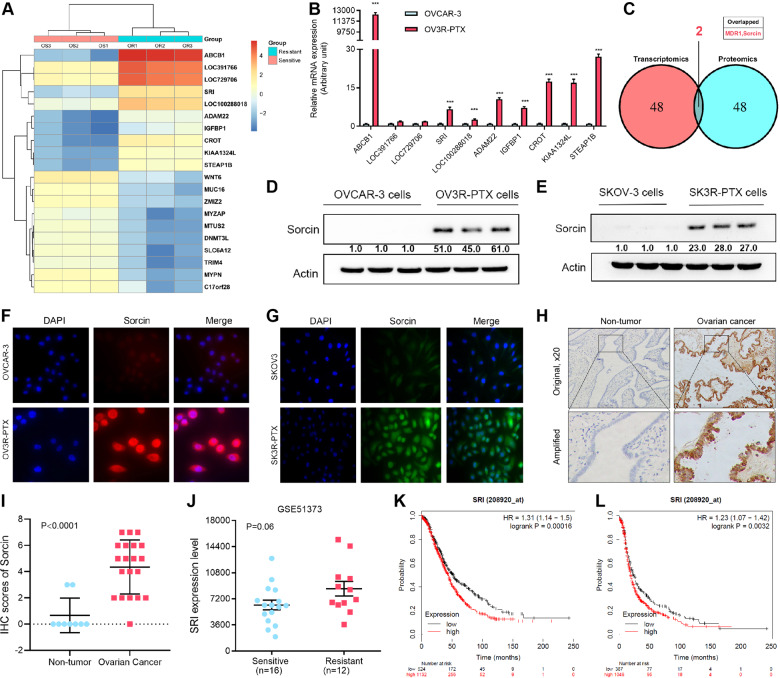
Expression of sorcin (SRI) in PTX-resistant ovarian cancer. **A** Heatmap showed the top ten upregulated and downregulated mRNAs in paclitaxel-resistant OV3R-PTX cells compared with paclitaxel-sensitive OVCAR-3 cells by a cutoff value of more than 2-fold. **B** Top ten upregulated mRNAs in the microarray were confirmed by qRT-PCR. ***, *p* < 0.001. **C** Analysis of the top 50 upregulated mRNAs and proteins from transcriptomes and proteomics. SRI and MDR1 were overexpressed both at mRNA and protein levels in OV3R-PTX cells. **D** SRI protein expression in OVCAR-3 and OV3R-PTX cells detected by Western blot. **E** SRI protein expression in SKOV-3 and SK3R-PTX cells detected by Western blot. **F** Immunofluorescence staining of SRI in OVCAR-3 and OV3R-PTX cells. **G** Immunofluorescence staining of SRI in SKOV3 and SK3R-PTX cells. **H** Representative images of SRI protein expression in primary ovarian cancer and non-tumorous ovarian tissue by immunohistochemistry. Original magnification, ×20. **I** Comparison of SRI protein expression between non-tumorous ovarian tissues (*n* = 9) and OC tissues (*n* = 20). **J** Comparison of SRI mRNA expression between chemotherapy-sensitive (*n* = 16) and chemotherapy-resistant (*n* = 12) ovarian serous carcinoma. **K** Kaplan–Meier analysis of the overall survival of OC patients associated with SRI expression. **L** Kaplan–Meier analysis of the progression-free survival of OC patients associated with SRI expression.

Transcriptome microarray showed the top ten upregulated and downregulated mRNAs in OV3R-PTX cells compared with OVCAR-3 cells by a cutoff value of more than 2-fold (Fig. 1A), which were confirmed by a qRT-PCR (Fig. 1B and Fig. S1A). Further analyses of the top 50 upregulated genes and proteins in transcriptomes and mass spectrometry of cell lysates showed only SRI and ABCB1 (MDR1) overexpression at both mRNA and protein levels in OV3R-PTX cells compared with OVCAR-3 cells (Fig. 1C and Supplementary Table S1), indicating that SRI was co-amplified with ABCB1. Next, we focused on SRI since little is known about SRI in PTX-resistant OC. The expression of SRI mRNA and protein was higher in OC cells compared to normal ovarian surface epithelial cells (HOSEpiC) detected by qRT-PCR and Western blot, respectively (Fig. S1B and S1C). Further, we found that the expression of SRI protein was much higher in PTX-resistant cells (OV3R-PTX and SK3R-PTX) compared with PTX-sensitive cells (OVCAR-3 and SKOV-3) detected by Western blot (Fig. 1D, E) and IF (Fig. 1F, G). Indeed, SRI was overexpressed in human OC tissues compared with non-tumor ovarian tissues detected by IHC (Fig. 1H). Semi-quantitative analysis of IHC showed that the level of SRI protein expression was higher in OC tissue than in non-tumor tissue (*p* < 0.0001; Fig. 1I). Public dataset mining further displayed that there was a trend in SRI appearing higher in chemoresistant tumors as compared to sensitive ones (*p* = 0.06; Fig. 1J). Furthermore, OC patients with a high SRI expression had a worse overall survival and progression-free survival (Fig. 1K, L).

### Sorcin promotes OC cell proliferation, migration, invasion, and EMT

**Fig. 2:**
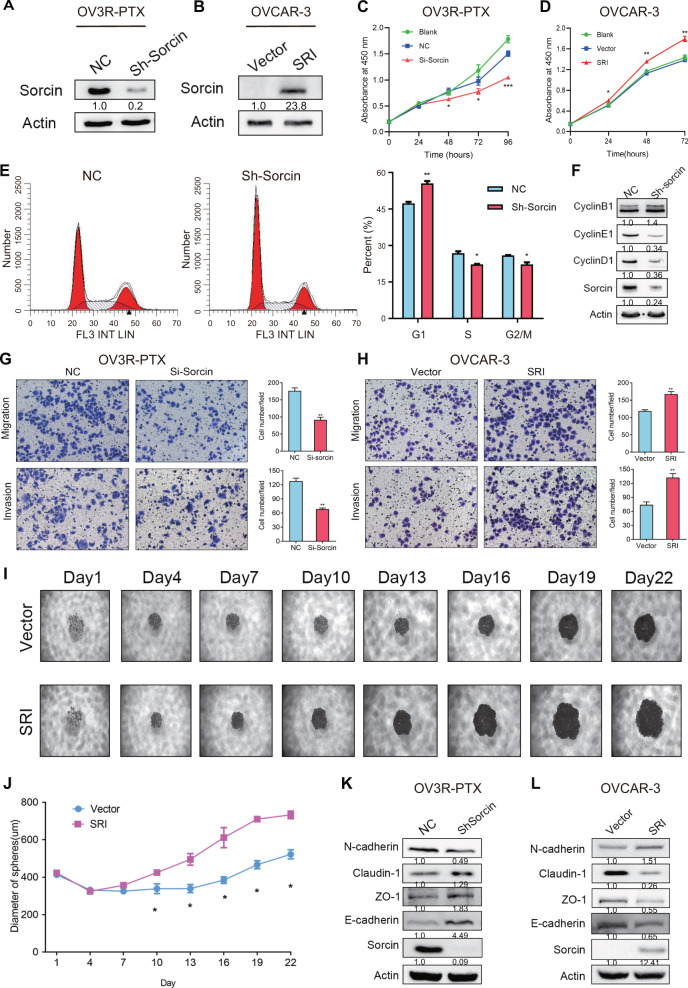
Effect of sorcin (SRI) on cell proliferation, migration, invasion, and EMT. **A** SRI protein expression in OV3R-PTX cells after Sh-sorcin infection detected by Western blot. **B** SRI protein expression in OVCAR-3 cells after SRI plasmid transfection detected by Western blot. **C** OV3R-PTX cell proliferation after Si-Sorcin infection detected by the CCK8 assay. **D** OVCAR-3 cell proliferation after SRI plasmid transfection detected by the CCK8 assay. **e** Cell cycle detection in OV3R-PTX cells after Sh-Sorcin infection by flow cytometry. The histograms show the percentage of the cell population in each phase. **F** Expression of CyclinB1, CyclinD1, and CyclinE1 in Sh-sorcin-infected OV3R-PTX cells detected by Western blot. **G** Migration and invasion of OV3R-PTX cell after Sh-Sorcin infection for 48 h. **H** Migration and invasion of OVCAR-3 cell after SRI plasmid transfection for 48 h. Scale bar, 100 µm. **I** Representative images of multicellular tumor spheroids with SRI overexpression in OVCAR-3 cells. Pictures were taken by phase-contrast microscopy every 3 days started from day 1. Original magnification, ×100. **J** Quantitative analysis of spheroid diameter from (**i**) (*n* = 3 independent experiments). **K** Expression of EMT marker N-cadherin, Claudin-1, ZO-1, and E-cadherin in Sh-Sorcin infected OV3R-PTX cells detected by Western blot. **L** Expression of N-cadherin, Claudin-1, ZO-1, and E-cadherin in SRI plasmid transfected OVCAR-3 cells detected by Western blot. NC, negative control; *, *p* < 0.05; **, *p* < 0.01; ***, *p* < 0.001.

To elucidate the biological function of SRI in OC, three SRI-specific siRNAs were designed to silence SRI expression. The efficiency of SRI knockdown was shown in (Fig. S2A). The sequence for the best knockdown of SRI expression was selected to generate the SRI-shRNA and an SRI-overexpressing plasmid. The knockdown and overexpression of SRI in OV3R-PTX and OVCAR-3 cells, respectively, were confirmed by Western blot (Fig. 2A, B). The knockdown and overexpression of SRI significantly decreased and increased proliferation in PTX-resistant cells (OV3R-PTX and SK3R-PTX) and PTX-sensitive cells (OVCAR-3 and SK-OV-3), respectively (Fig. 2C, D; Fig. S2B and S2C). The flow cytometry analysis showed that the knockdown of SRI arrested the OV3R-PTX cell cycle at the G1 phase (Fig. 2E). Western blot also showed a decrease in the expression of G1 phase-related cyclins (Cyclin D1 and Cyclin E1) but not the Cyclin B1 after SRI knockdown (Fig. 2F). The knockdown of SRI resulted in a decrease in cell migration and invasion in OV3R-PTX and SK3R-PTX cells (Fig. 2G and Fig. S3A), whereas the overexpression of SRI resulted in an increase in cell migration and invasion in OVCAR-3 and SKOV-3 cells (Fig. 2H and Fig. S3B). Further studies using a three-dimensional cell culture system showed that the knockdown and overexpression of SRI decreased and increased the size of a tumor spheroid in OV3R-PTX and OVCAR-3 cells, respectively (Fig. S3C and S3D; Fig. 2I and J). Intriguingly, GSEA and cell morphology analyses revealed that SRI participated in the establishment of cell polarity and EMT (Fig. S4A and S4B). The expression of N-cadherin was decreased, whereas Claudin-1, ZO-1, and E-cadherin levels were increased, after SRI-shRNA infection in OV3R-PTX cells (Fig. 2K) and an opposite effect was observed after overexpression of SRI in OVCAR-3 cells (Fig. 2L). Furthermore, IF staining with E-cadherin and N-cadherin confirmed the above results (Fig. S4C and S4D).

### Sorcin is involved in stemness, tumor growth, and metastasis

**Fig. 3:**
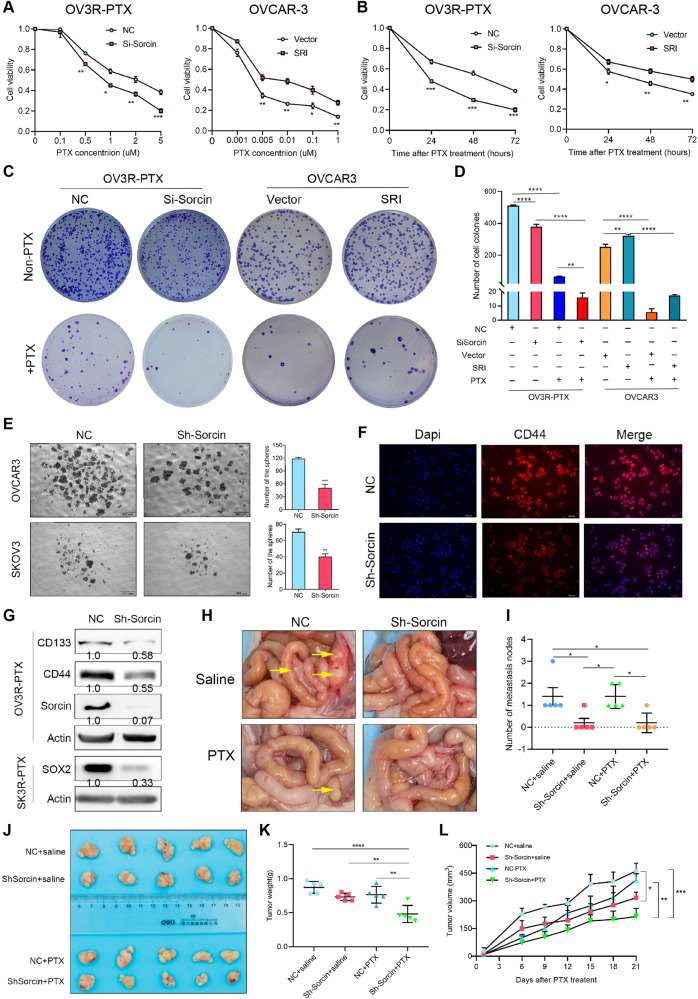
Effect of sorcin (SRI) on paclitaxel sensitivity, stemness, and metastasis in vitro and in vivo. **A** Cell viability of OV3R-PTX after SRI knockdown and OVCAR-3 after SRI overexpression in the presence of different doses of PTX for 2 days detected by the CCK-8 assay. **B** Cell viability of OV3R-PTX after SRI knockdown in the presence of 5 μM PTX and OVCAR-3 after SRI overexpression in the presence of 0.01 μM PTX for different days detected by the CCK-8 assay. **C** Colony formation of SRI-shRNA-infected OV3R-PTX cells with or without 5 μM PTX treatment and SRI plasmid-transfected OVCAR-3 cells with or without 0.01 μM PTX treatment. **D** Quantification of colony formation assays in (**C**). **E** Spheroid formation of OC cells after SRI-shRNA infection for 14 days. Original magnification, ×40; scale bar, 500 µm. **F** Immunofluorescence staining of CD44 in SRI-shRNA infected OVCAR-3 cells. Original magnification, ×200; scale bar, 100 µm. **G** Expression of CD133, CD44, SOX2, and sorcin expression in OVCAR-3 cells after sorcin knockdown detected by Western blot. **H** Representative images of the abdomen metastatic foci in nude mice after intraperitoneally injected with NC or Sh-Sorcin-infected OV3R-PTX cells in the presence or absence of PTX. **I** Number of metastatic nodes (*n* = 5/group). **J** Xenograft tumor formation in nude mice. NC or Sh-Sorcin-infected OV3R-PTX cells were subcutaneously implanted in the presence or absence of PTX (*n* = 5/group). **K** Measurement of tumor weight from (**J**). **L** Measurement of tumor size from (**J**). NC negative control; *, *p* < 0.05; **, *p* < 0.01; ***, *p* < 0.001.

The knockdown of SRI expression effectively increased the sensitivity or decreased the resistance of OV3R-PTX cells to PTX, while overexpression of SRI significantly decreased the sensitivity or increased the resistance of OVCAR-3 cell to PTX, in dose-dependent (Fig. 3A) and time-course assays (Fig. 3B). In colony formation assay, knockdown of SRI expression decreased the capacity of colony formation in OV3R-PTX cells with or without 5 μM PTX treatment and increased PTX sensitivity, while overexpression of SRI had an opposite effect in OVCAR-3 cells (Fig. 3C, D).

Since cancer stem cells (CSCs) were believed to be the main drivers of tumorigenesis, recurrence, chemoresistance, and metastasis, we performed the GSEA analysis by using data from our transcriptome microarray (Accession #GSE168927). Significant enrichment of genes in the regulation of stem cell differentiation was associated with different expression levels of SRI (Fig. S5A). The spheroid formation showed that the downregulation of SRI inhibited the ability of sphere formation in two OC cell lines (Fig. 3E). IF staining confirmed the decrease of stemness by the detection of CD44 expression after SRI knockdown (Fig. 3F), which was further validated by Western blot for stemness markers CD133, CD44, and SOX2 (Fig. 3G), indicating that SRI affects OC stemness.

The in vivo studies of the tumor xenograft and metastatic models were applied. The knockdown of SRI significantly inhibited the abdomen tumor foci and reduced peritoneal metastatic nodules, but which were not affected by PTX treatment (Fig. 3H, I). Next, the tumor xenograft assay showed that tumor size was significantly smaller by reducing the tumor weight and volume in SRI-shRNA-infected OV3R-PTX cells after PTX treatment (Fig. 3J–L).

### Sorcin is negatively regulated by miR-142-5p

**Fig. 4:**
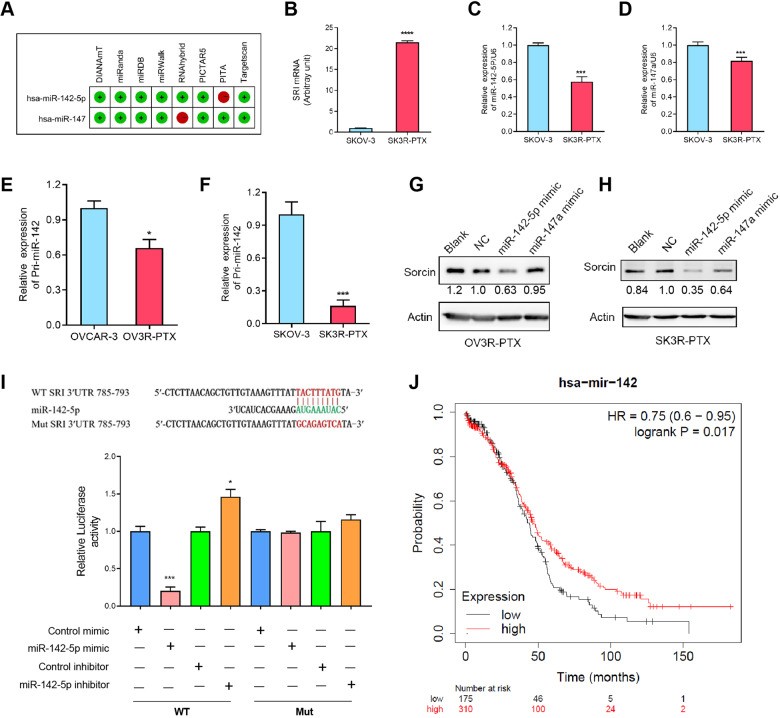
Effect of miR-142-5p on sorcin (SRI) expression. **A** Prediction of miRNAs binding to SRI. miR-142-5p and miR-147a were predicted as the most likely candidates for SRI by seven out of eight miRNA-associated prediction programs. **B** Expression of SRI mRNA in SKOV-3 and SK3R-PTX cells detected by qRT-PCR. **C** Expression of miR-142-5p in SKOV-3 and SK3R-PTX cells detected by qRT-PCR. **D** Expression of miR-147a in SKOV-3 and SK3R-PTX cells detected by qRT-PCR. e Expression of Pri-miR-142 in OVCAR-3 and OV3R-PTX cells detected by qRT-PCR. **F** Expression of Pri-miR-142 in SKOV-3 and SK3R-PTX cells detected by qRT-PCR. **G** Expression of sorcin in OV3R-PTX cells transfected with miR-142-5p and miR-147a mimics detected by Western blot. **H** Expression of sorcin in SK3R-PTX cells transfected with miR-142-5p or miR-147a mimics detected by Western blot. **I** Partial sequence of SRI 3ʹ-UTR. The wild-type (WT) and mutated (Mut) 3ʹ-UTR sequences of hsa-miR-142-5p were shown at the top. Nucleotides in red in WT SRI 3ʹUTR indicated complementarity to miR-142-5p and were mutated in Mut SRI 3ʹ-UTR plasmid. Relative luciferase activities in HEK 293 T cells treated with a miR-142-5p mimic or inhibitor were shown at the bottom. **J** Kaplan–Meier analysis of the overall survival of OC patients associated with has-miR-142 expression. Blank, cells without transfection; NC negative control; *, *p* < 0.05, ***, *p* < 0.001.

Using the miRWalk1.0 database, we found that miR-142-5p and miR-147a were the most likely candidates for SRI by 7 out of 8 miRNA-associated prediction programs (Fig. 4a). A high level of SRI mRNA was observed in SK3R-PTX cells compared with SK-OV-3 cells (Fig. 4B), whereas the low levels of miR-142-5p and miR-147a expression were observed in SK3R-PTX compared with SK-OV-3 cells by qRT-PCR (Fig. 4C, D). Since miR-142-5p had more efficiency than miR-147a to decrease the expression of Sorcin, we chose miR-142-5p in the following studies. Further analysis showed that primary miR-142 expression was lower in two chemoresistant cells (OV3R-PTX and SK3R-PTX) than their counterpart sensitive cells (Fig. 4E, F). The transfection of miR-142-5p mimics significantly inhibited SRI expression in both OV3R-PTX and SK3R-PTX cells (Fig. 4G, H). To determine whether miR-142-5p directly binds to the 3ʹ-UTR of SRI, we constructed a wild-type (SRI-3UTR-wt) and a mutated (SRI-3UTR-mut) SRI 3ʹ-UTR plasmids. Ectopic expression of miR-142-5p dramatically decreased the luciferase activity, whereas the inhibition of miR-142-5p by its inhibitor increased the luciferase activity in the cells expressing SRI-3UTR-wt plasmids (Fig. 4I). However, miR-142-5p mimics or inhibitors no longer influenced the luciferase activity in the presence of the mutant plasmids. Furthermore, the Kaplan-Meyer analysis showed that low expression of miR-142 resulted in a worse overall survival of patients with OC (Fig. 4J).

### miR-142-5p affects cellular behavior that is partially abolished by sorcin

**Fig. 5:**
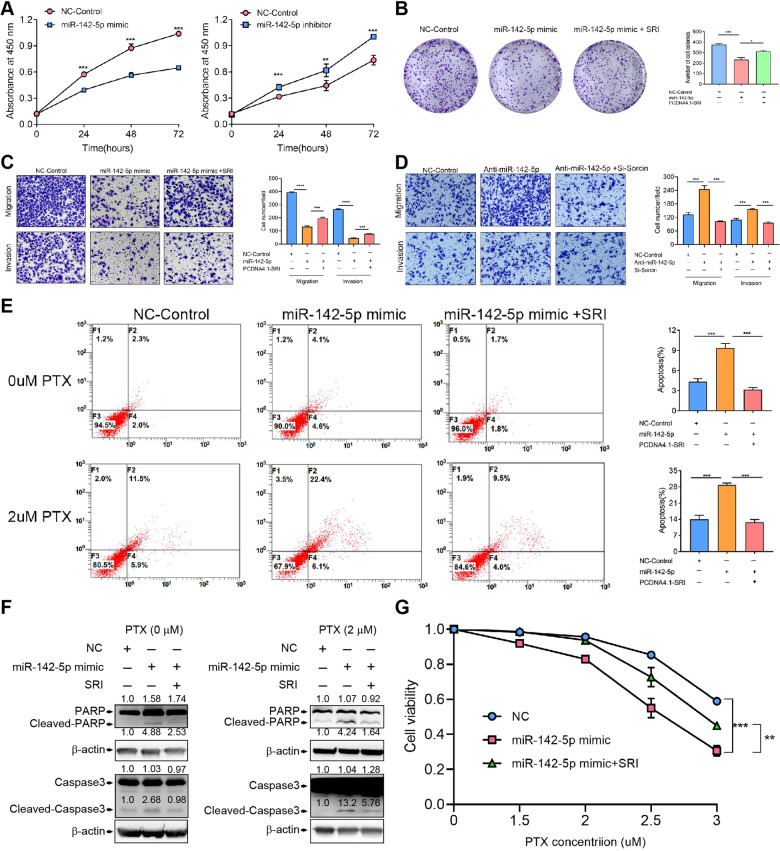
Effect of miR-142-5p on cellular behavior. **A** Cell proliferation of SK3R-PTX with miR-142-5p mimic or inhibitor detected by the CCK8 assay. **B** Colony formation of SK3R-PTX cells transfected with control, miR-142-5p mimic, or miR-142-5p plus SRI-overexpressing plasmid. **C** Migration and invasion of SK3R-PTX cells transfected with control, miR-142-5p mimic, or miR-142-5p plus SRI-overexpressing plasmid. Scale bar, 100 µm. **D** Migration and invasion of SK3R-PTX cells transfected with control, miR-142-5p inhibitor, or miR-142-5p inhibitor plus SRI-siRNA. Scale bar, 100 µm. **E** Detection of apoptotic cells by flow cytometry. SK3R-PTX cells were transfected control, miR-142-5p mimic, or miR-142-5p plus SRI-overexpressing plasmid in the presence or absence of 2 μM paclitaxel (PTX). **F** Expression of apoptotic markers PARP, cleaved-PARP, Caspase3, and Cleaved-Caspase3 in SK3R-PTX cells transfected with control, miR-142-5p mimic, or miR-142-5p plus SRI-overexpressing plasmid detected by Western blot. **G** Cell viability of SK3R-PTX transfected with control, miR-142-5p mimic, or miR-142-5p plus SRI-overexpressing plasmid after PTX treatment for 2 days. NC negative control; *, *p* < 0.05; **, *p* < 0.01; ***, *p* < 0.001.

The effect of miR-142-5p on SK3R-PTX cell proliferation was detected by the CCK-8 assay. miR-142-5p mimic significantly decreased, whereas the miR-142-5p inhibitor increased, cell proliferation in a time-course manner (Fig. 5A). Flow cytometry analysis revealed that miR-142-5p mimics arrested the cell cycle at the S phase (Fig. S5B). Transfection of miR-142-5p mimics significantly reduced the ability of colony formation; however, this inhibitory effect was partially reversed upon co-transfection with the SRI-overexpressing plasmid (Fig. 5B). Transwell assays showed that miR-142-5p mimics inhibited cell migration and invasion, which were partially reversed after transfection with the SRI-overexpressing plasmid (Fig. 5C). An opposite effect was observed in the presence of miR-142-5p inhibitor and SRI-siRNA (Fig. 5D). Flow cytometry revealed that SRI-siRNA induced apoptosis and more apoptotic cells were observed after PTX treatment, whereas overexpression of SRI had the opposite effect (Fig. S6A and S6B). SRI-siRNA-induced apoptosis was confirmed by the detection of apoptotic protein cleaved-caspase 3 after 1 μM PTX treatment (Fig. S6C). Interestingly, miR-142-5p mimics induced cell apoptosis, which was abolished in the SRI-overexpressing cells, and even in the presence of 2 μM PTX (Fig. 5E). These results were further confirmed by the detection of cleaved-PARP and cleaved-caspase 3 after the treatment of miR-142-5p mimics and PTX, which was partially blocked in SRI-overexpressing cells (Fig. 5F). These results indicate that PTX and miR-142-5p were synergized in promoting PTX-resistant cell apoptosis. Furthermore, cell viability assays showed that miR-142-5p mimics increased the sensitivity of PTX in resistance cells, while overexpression of SRI blocked this effect in a dose-dependent manner (Fig. 5G). These data indicate that SRI is a downstream molecule of miR-142-5p.

### ZEB1 represses miR-142-5p expression to mediate sorcin expression

**Fig. 6:**
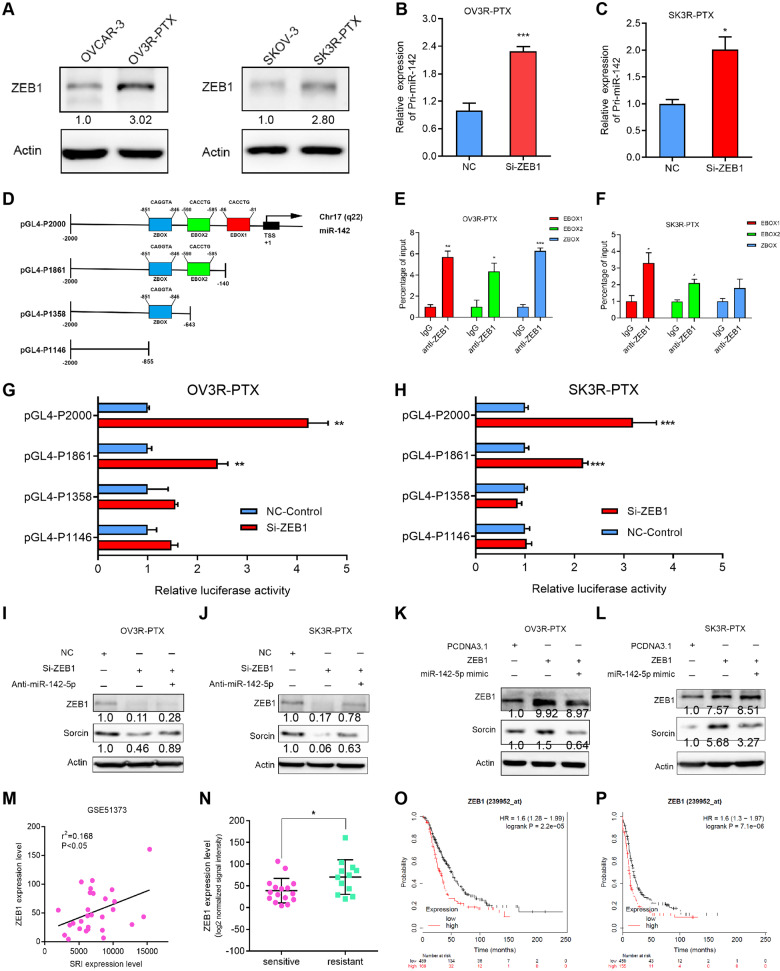
Regulation of ZEB1 on miR-142-5p expression. **A** ZEB1 protein expression in OVCAR-3, OV3R-PTX, SKOV-3, and SK3R-PTX cells detected by Western blot. **B** Expression of Pri-miR-142 in the negative control (NC)-siRNA or ZEB1-siRNA-transfected OV3R-PTX cells by qRT-PCR. **C** Expression of Pri-miR-142 in NC or ZEB1-siRNA-transfected SK3R-PTX cells by qRT-PCR. **D** Schematic illustrations of 4 constructs: a full promoter region pGL4-P2000 and three truncated promoter regions pGL4-P1861, pGL4-P1358, and pGL4-P1146. **E** ChIP-qPCR assay for ZEB1 binding to the miR-142 promoter in OV3R-PTX cells. **F** ChIP-qPCR assay for ZEB1 binding to the miR-142 promoter in SK3R-PTX cells. **G** Luciferase activities of different miR-142 promoter-reporter constructs with co-transfection of ZEB1-siRNA or NC in OV3R-PTX cells. **H** Luciferase activities of different miR-142 promoter-reporter constructs with co-transfection of ZEB1-siRNA or NC in SK3R-PTX cells. **I** Sorcin expression in OV3R-PTX cells after transfection of NC, ZEB1-siRNA, or miR-142-5p inhibitor plus ZEB1-siRNA detected by Western blot. **J** Sorcin expression in SK3R-PTX cells after transfection of NC, ZEB1-siRNA, or miR-142-5p inhibitor plus ZEB1-siRNA detected by Western blot. **K** Sorcin expression in OV3R-PTX cells after transfection of pcDNA3.1 vector, ZEB1-overexpressing plasmid, or miR-142-5p mimics plus ZEB1-overexpressing plasmid detected by Western blot. **L** Sorcin expression in SK3R-PTX cells after transfection of pcDNA3.1 vector, ZEB1-overexpressing plasmid, or miR-142-5p mimics plus ZEB1-overexpressing plasmid detected by Western blot. **M** Correlation analysis of SRI and ZEB1 mRNA expression in the GSE51373 dataset. **N** Comparison of ZEB1 mRNA expression between chemotherapy-sensitive (*n* = 12) and chemotherapy-resistant (*n* = 16) ovarian serous carcinoma from the GSE51373 dataset. **O** Kaplan–Meier analysis of the overall survival of OC patients associated with ZEB1 expression. **P** Kaplan–Meier analysis of the progression-free survival of OC patients associated with ZEB1 expression. *, *p* < 0.05; **, *p* < 0.01; ***, *p* < 0.001.

To explore the underlying mechanism responsible for miR-142-5p expression in PTX-resistant OC cells, we searched the UCSC Genome Browser database combining with the JASPAR database to identify transcription factors that would bind to the miR-142-5p promoter region (2 kb upstream of the transcriptional start site) (Fig. S7A). ZEB1, one of the transcription factors found for miR-142-5p, was overexpressed in our two PTX-resistant cells (OV3R-PTX and SK3R-PTX) (Fig. 6A). The knockdown of ZEB1 increased pri-miR-142 expression in OV3R-PTX and SK3R-PTX (Fig. 6B, C). ZEB1 is a member of the zinc finger-type transcription factors and can bind to the promoter DNA of a gene containing CACCTG or CAGGTG (E-box)) and CAGGTA (Z-box) binding motifs [24]. miR-142 promoter regions without or with 1–3 ZEB1-binding sites were then cloned into the pGL4 vector to generate four expressing plasmids, including a full promoter region pGL4-P2000 (-2000/-1 upstream sequence) and three truncated promoter regions (P1861, P1358, and P1146) (Fig. 6D). Using a ChIP-qPCR assay, we found that ZEB1 bound to the E-box1 and E-box2 sites of miR-142 in OV3R-PTX and SK3R-PTX cells (Fig. 6E, F). Negative control of ChIP-qPCR assay was shown in Fig. S7B. Furthermore, the dual-luciferase assay showed that silencing ZEB1 by ZEB1-siRNA increased the luciferase activity in the presence of P2000 and P1861, whereas no activity was observed in the presence of P1358 and P1146 in OV3R-PTX and SK3R-PTX (Fig. 6G, H). Next, we confirmed ZEB1 directly binding to miR-142-5p to regulate SRI expression. Silencing ZEB1 resulted in a decrease of SRI expression which was partially abolished by miR-142-5p inhibitor in OV3R-PTX and SK3R-PTX (Fig. 6I, J), whereas overexpression of ZEB1 increased SRI expression which was partially abolished by miR-142-5p mimics in OV3R-PTX and SK3R-PTX (Fig. 6K, L). The positive correlation between ZEB1 and SRI was further confirmed in the GSE51373 dataset using bioinformatics analysis (Fig. 6M). Furthermore, the expression of ZEB1 was higher in chemoresistant patients than chemosensitive patients with OC in the GSE51373 dataset (Fig. 6N). Finally, the Kaplan-Meyer analysis revealed that OC patients with high ZEB expression had a worse overall survival and progression-free survival (Fig. 6O, P).

### Sorcin interacts with Smad4 and regulates ZEB1 expression

**Fig. 7:**
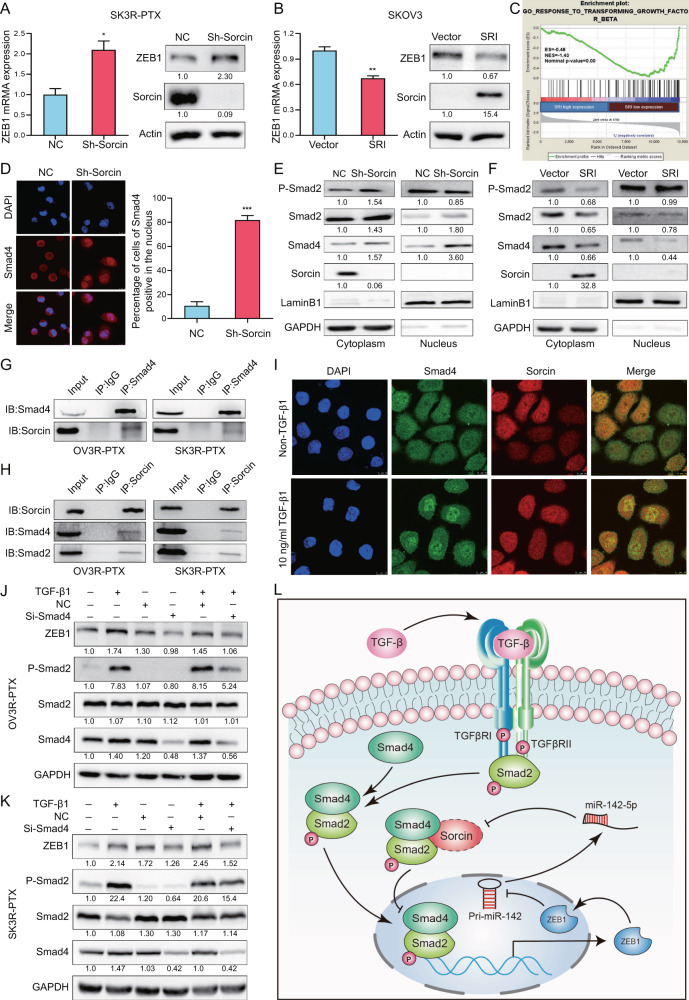
Effect of Sorcin (SRI) on ZEB1 and Smads in PTX-resistant OC cells. **A** ZEB1 mRNA and protein expression in SRI-shRNA-infected OV3R-PTX cells detected by qRT-PCT and Western blot, respectively. **B** ZEB1 mRNA and protein expression in SRI-plasmid-transfected OVCAR-3 cells detected by qRT-PCT and Western blot. **C** Gene Set Enrichment Analysis (GSEA) of significant enrichment in the genes regulated by transforming growth factor-β (TGF-β) with different expression levels of SRI. **D** Immunofluerensce staining of Smad4 in SK3R-PTX cells after SRI-shRNA infection. The cells of Smad4-positive in the nucleus were counted at least in 3 fields and a percentage of Smad4-positive cells was shown. **E** Detection of phospho-Smad 2, Smad2, Smad4, and SRI proteins from the cytosol and nucleus of SRI-shRNA-infected SK3R-PTX cells by Western blot. GAPDH and Lamin B1 served as cytoplasmic and nuclear protein loading controls, respectively. **F** Detection of phospho-Smad 2, Smad2, Smad4, and SRI proteins from the cytosol and nucleus of SRI plasmid-transfected OVCAR-3 cells by Western blot. **G** Co-immunoprecipitation of Smad4 with SRI in OV3R-PTX and SK3R-PTX cells. Smad4 was immunoprecipitated with an anti-Smad4 antibody or IgG and analyzed by Western blot with anti-Smad4 and anti-SRI antibodies. **H** Co-immunoprecipitation of SRI with Smad4 and Smad2 in OV3R-PTX and SK3R-PTX cells. SRI was immunoprecipitated with an anti-SRI antibody or IgG and analyzed by Western blot with anti-SRI and anti-Smad4 antibodies. **I** Detection of Smad4 and SRI co-localization in OV3R-PTX cells in the presence or absence of 10 ng/μl TGF-β1 by confocal microscopy. Alexa Fluor 488 (green) or Alexa Fluor 594 (red) was used to detect Smad4 and SRI, respectively. The images of Smad4 and SRI were merged. The nucleus was stained with DAPI. **J** Effect of TGF-β/Smad4 on ZEB1 expression in OV3R-PTX cells. **K** Effect of TGF-β/Smad4 on ZEB1 expression in SK3R-PTX cells. **L** Schematic illustration of the regulatory mechanism of a Sorcin/Smad4/ZEB1/miR-142-5p homeostatic loop in paclitaxel-resistant ovarian cancer cells. The dashed lines of the displayed SRI indicated that the interaction of SRI with Smad complex might be weak and/or transient.

Silencing SRI increased, whereas overexpressing SRI decreased, ZEB1 expression at mRNA and protein levels (Fig. 7A, B). By analyzing data from our transcriptome microarray using the GSEA, we found that the level of SRI expression was negatively correlated with genes response to transforming growth factor-β (TGF-β) (Fig. 7C). Indeed, phospho-Smad2 (P-Smad2) and Smad4 negatively correlated with SRI expression in chemosensitive and chemoresistant OC cells. High levels of SRI expression with low levels of P-Smad2 and Smad4 were found in OV3R-PTX and SK3R-PTX cells compared to their counterpart sensitive cells (Fig. S8A). The low level of Smad4 was further confirmed in chemoresistant OC patients from the GSE51373 dataset (Fig. S8B). Interestingly, knockdown of SRI resulted in the nuclear accumulation of Smad4 detected by immunofluorescence staining (Fig. 7D). Knockdown of SRI slightly increased P-Smad2 in the cytosolic fraction and Smad2 and Smad4 in the nuclear fraction of SK3R-PTX cells (Fig. 7E), In contrast, overexpression of SRI decreased P-Smad2 in the cytosolic fraction and reduced Smad2 and Smad4 in the nuclear fraction of OVCAR-3 cells (Fig. 7F). Co-immunoprecipitation demonstrated that SRI physically interacted with Smad4/2 in chemoresistant OV3R-PTX and SK3R-PTX cells though the signals in co-IP were weak than input ones (Fig. 7G, H), indicating that the interaction of SRI with Smad complex might be weak and/or transient. Upon TGF-β1 stimulation, the co-localization of SRI and Smad4 in the cytosol and the nuclear translocation of Smad4 in OV3R-PTX and SK3R-PTX cells were observed by confocal microscopy (Fig. 7I and Fig. S8C). Furthermore, TGF-β1 increased ZEB1 expression, whereas knockdown of Smad4 by Smad4-siRNA decreased ZEB1 expression and partially abolished the effect of TGF-β1 on ZEB1 expression in two PTX-resistant cell lines (Fig. 7J, K). The phosphorylation of Smad2 was induced upon TGF-β1 treatment, indicating that the TGF-β/Smad signaling pathway was intact in these cells.

## Discussion

The current study demonstrated that SRI as a PTX resistance-related factor was upregulated in OC and was correlated with the overall survival of patients with OC. Overexpressed SRI modulated biological behavior and stem-like cell phenotype in OC cells. Mechanistically, SRI was found to be a direct target of miR-142-5p that mediated proliferation, migration, invasion, EMT, apoptosis, and PTX sensitivity. miR-142-5p was transcriptionally repressed by a transcription factor ZEB1 which was mediated by SRI and the TGF-β signaling pathway.

Our previous study has shown that OC cell stemness properties are associated with PTX-resistance [25]. Studies from other groups have shown that SRI is a chemoresistance-related protein overexpressed in lung, breast, gastric, and colorectal cancer cell lines, etc [10, 11, 26–28]. Although a previous study showed the transfection of full-length SRI cDNA inducing PTX resistance in ovarian and breast cancer cells [29], the interpretation of observation is not clear. The current demonstrated that SRI was indeed a PTX-resistant protein and could regulate the cancer stem cell-like characteristics of OC cells. Silencing SRI resulted in a decrease in the cancer stem cell markers CD133, CD44, and SOX2. Accumulating evidence indicates that the chemoresistant tumor cells are closely related to cancer stem cells [30, 31], suggesting that cancer stem cells are believed to be the main cause of tumorigenesis, recurrence, and chemoresistance in OC.

It has been shown that SRI can reduce cancer stem cell subpopulation and acts as an oncoprotein to regulate EMT in breast cancer [8]. EMT has been strongly associated with chemoresistance and the acquisition of drug resistance, in turn, promotes the abnormal activation of tumor cells [30, 32]. The present study unveiled the promotion of EMT by SRI in OC cells, indicating that SRI may be associated with the malignant progression and development of OC that may affect OC patient outcomes. Indeed, our Kaplan–Meier survival analysis found the high expression of SRI correlated with a worse clinical prognosis in patients with OC. Because resistant cancer cells gradually lose their ability to undergo programmed cell death leading to uncontrolled proliferation [33] and because the knockdown of SRI combined with PTX treatment increased cell apoptosis and reduced colony formation in the current study, we speculate that target SRI may reverse the PTX-resistance and regain the sensitivity of OC patients to PTX.

Most notable in the current mechanistic study is that SRI was suppressed by miR-142-5p which was downregulated in OC patients. miR-142-5p directly bound to the 3ʹ-UTR of SRI, suggesting that SRI was essential for miR-142-5p-induced migration, invasion, apoptosis, colonization abilities, and PTX resistance in OC cells. Our study further demonstrated that ZEB1 transcriptionally inhibited miR-142-5p expression by directly binding to the E-box fragment in the miR-142 promoter region. It has been reported that transcription factors act as drivers of cancer initiation and progression [34]. The initiation of metastasis requires EMT which is mediated by several transcription factors, including ZEB1. Aberrant expression of ZEB1 has been reported to be involved in chemoresistance in several types of cancer, including lung, breast, prostate, and liver cancers [35–38]. Here, we demonstrated that ZEB1 was upregulated in PTX-resistant OC cells and chemoresistant patients and was positively correlated with SRI expression. Furthermore, bioinformatics analyses of the public database showed that ZEB1 expression was inversely correlated with survival outcomes. Indeed, ZEB1 can induce EMT-activation and stemness by directly inhibiting several miRNAs and thereby form a feedback loop in mediating tumor development [39]. Our mechanistic studies indicated that the ZEB1/miR142-5p/Sorcin axis might exist.

A previous study reported that TGF-β can induce the mesenchymal phenotype, stabilize a stem cell-like state, and promote anticancer drug resistance in mammary epithelial and carcinoma cells [40]. It has been shown that the expression of EMT-related transcription factors is activated either through a directly Smad-dependent mechanism or indirect activation of other transcription factors upon TGF-β treatment [41]. Indeed, TGF-β-induced EMT via a transcription factor ZEB1 is complex. ZEB1 is a downstream molecule of TGF-β and is necessary, but not sufficient, for TGF-β-induced EMT [42]. ZEB1 can interact with Smads and form EMT-promoting Smad complexes (EPSC) to both repress epithelial genes and activate mesenchymal genes [43]. On the other hand, the loss of Smad4 (a transducer of TGF-β signaling) promotes cancer progression and induces EMT [44]. In the current study, we observed that SRI physically interacting with Smad4 and blocked Smad4 nuclear translocation, indicating that SRI-induced EMT may be via the Smad4 pathway. Furthermore, ZEB1 expression was suppressed and regulated by SRI, suggesting that there is a feedback loop of SRI/Smad4/ZEB1/miR-142-5p.

The current study may endorse an alternative way for the treatment of OC patients with PTX-resistance. The dysregulation of the SRI/Smad4/ZEB1/miR-142-5p loop leads to PTX-resistance and the intervention of this axis may be a good therapeutic strategy. The previous report shows that SRI has a possible prognostic value in childhood lymphoblastic leukemia [45], while inactivation of Smad4 results in drug resistance in colon cancer [44]. Targeting SRI by a miRNA has also been reported. For instance, miR-1 inhibits SRI expression in gastric cancer [9]. Some groups show that natural compounds such as dihydromyricetin and triptolide can be able to reverse drug resistance by decreasing SRI expression in vitro [46, 47], indicating that SRI is most likely a good targeting candidate to reverse or prevent chemoresistance. Our study also showed for the first time that miR-142-5p affected PTX-resistance and cellular behavior partially through regulating SRI expression, suggesting that miR-142-5p mimic may have a potential application in OC treatment. Indeed, the therapeutic potential of miR-142-5p for gefitinib resistance in lung cancer cells is reported [48]. The current work indicated that ZEB1 functions as a transcription factor of miR-142-5p, while data from another group indicated that ZEB1 is involved in PTX-resistance in prostate cancer [49]. Thus, based on these studies, we propose a therapeutic strategy by targeting this loop in OC.

In summary, SRI is overexpressed in PTX-resistant OC cells and is related to the poor prognosis of patients, acting as a PTX-resistant protein involved in cell proliferation, EMT, stemness, and PTX-resistance. miR-142-5p directly binds to the 3ʹ-UTR of SRI to suppress its expression, whereas ZEB1 inhibits the transcription of miR-142-5p by blocking its promotor E-box binding sites. ZEB1 is negatively regulated by SRI which transiently interacts with the Smad4 complex to block its translocation from the cytosol to the nucleus (Fig. 7l). Our findings unveil a novel homeostatic loop of SRI that drives the PTX-resistance and malignant progression via Smad4/ZEB1/miR-142-5p in human OC. Targeting this SRI/Smad4/ZEB1/miR-142-5p loop may reverse the PTX-resistance.

## Materials and methods

### Cell lines and cell culture

The human OC cell lines (OVCAR-3, SK-OV-3, Caov-3, A2780, ES-2) and non-tumorous human ovarian surface epithelial cells (HOSEpiC) have been described previously [50]. PTX-resistant OVCAR-3 cells (OV3R-PTX) [25] and SK-OV-3 cells (SK3R-PTX) were generated in our laboratory. The PTX-resistant SK3R-PTX cells were derived from parental SK-OV-3 cells by treating cells with the PTX regimen through a gradually increasing PTX dose in Dulbecco’s Modified Eagle’s Medium with 10% fetal bovine serum (FBS) (Invitrogen, Carlsbad, CA, USA) and 1% streptomycin/penicillin (Beyotime, Shanghai, China).

### Transfection of siRNA, miRNA, and plasmid and infection of shRNA lentivirus

Brief transfection and infection methods were provided in Supplementary Methods. All sequences are listed in Supplementary Table S2.

### Quantitative RT-PCR

Quantitative RT-PCR was performed as described previously [50]. Primer sequences are listed in Supplementary Table S3.

### Extraction of the total, cytoplasmic, and nuclear proteins and western blot

Protein was extracted and described in Supplementary Methods. Western blot analysis was performed as described previously [50]. All primary antibodies are listed in Supplementary Table S4.

### Immunohistochemical staining (IHC)

A total of 20 paraffin-embedded ovarian tumor tissues and 9 non-tumorous ovarian tissues were obtained from Jinshan Hospital, Fudan University. All patients had not received chemotherapy or radiotherapy before surgery. Ethics approval was approved by the Ethics Committee of Jinshan Hospital (JYLLKY-2019-01-01). IHC was performed as described previously [50] and Supplementary Methods.

### Immunofluorescence (IF) and confocal microscopy

IF and confocal microscopy were described in Supplementary Methods. All antibodies are listed in Supplementary Table S4.

### Migration, invasion, and proliferation assays

OV3R-PTX (5 × 10^4^) and SK3R-PTX (8 × 10^4^) cells were suspended in a serum-free medium and plated on the upper chamber. The bottom chamber was supplied with a medium containing 10% FBS. After 48 h, the migrant or invasive cells were counted as described previously [50]. Cell proliferation was measured by Cell Counting Kit-8 (CCK-8, Dojindo Laboratories, Kumanoto, Japan) assay.

### Three-dimensional cell culture

Agar gel (1%, Sigma) was plated on a 96-well plate. SRI-shRNA-infected cells and NC cells were seeded to each well at a density of 3000 cells/well. The growth characteristics of spheroids were recorded every 3 days using an inverted microscope (IX73; Olympus Corporation). The diameter (D) of a spheroid was calculated by the formula as described previously [51].

### Spheroid formation assay

Spheroid formation assay was performed as described previously [25] and Supplementary Methods.

### Chromatin immunoprecipitation (ChIP)-qPCR assay

ChIP was performed using the SimpleChIP® Enzymatic Chromatin IP Kit (Cell Signaling Technology) according to the manufacturer’s protocol. Briefly, SK3R-PTX cells were cross-linked with 1% PFA for 10 min at room temperature and terminated with glycine. Chromatin was sheared by micrococcal nuclease to generate DNA fragments in ≈150–900 bp. After incubation with ZEB1 antibody or control IgG (Cell Signaling Technology) at 4 °C overnight, immunoprecipitated DNA was eluted from antibody/protein G magnetic beads and reversal of cross-links. The immunoprecipitated DNA was detected by qPCR. Fold change was calculated based on Ct as 2^−ΔΔCT^, where ΔCt = Ct_IP_ − Ct_Input_ and (ΔΔCt) = ΔCt_antibody_ − ΔCt_IgG_. The primer sequences were listed in Supplementary Table S3.

### Protein co-immunoprecipitation

Proteins were extracted by centrifugation and incubated with an anti-Smad4 monoclonal antibody, an anti-SRI monoclonal antibody, or normal IgG control (Beyotime) (2 μg each) overnight at 4 °C with rotation, followed by the incubation of protein A + G Agarose (Beyotime) for 6 h. After boiling at 100 °C for 5 min, proteins were detected by SDS‐PAGE and Western blotting.

### Bioinformatics analysis

The expression level of SRI was assessed by Gene Set Enrichment Analysis (GSEA). A GSEA-4.0.0 software (http://software.broadinstitute.org/gsea/index.jsp) on various functional gene signatures was used to analyze our microarray profiling. A *P* value of <0.05 was set as the cutoff criteria. miRWalk (version 1.0; http://zmf.umm.uni-heidelberg.de/apps/zmf/mirwalk/predictedmirnagene.html) was used to predict the miRNAs targeting SRI. The University of California Santa Cruz (UCSC) genomic database (http://genome.ucsc.edu/) and the JASPAR database (http://jaspar.genereg.net/) were used to predict transcriptional factor ZEB1 binding sites in the miR-142 promoter region. The Kaplan–Meier plotter (www.kmplot.com) was applied to investigate the prognostic value of SRI, ZEB1, and miR-142 [52]. The cutoff value for an indicated gene is set as an auto-selected best cutoff expression level (high versus low expression). The expression data of GSE51373 for SRI, ZEB1, and Smad4 were obtained from the Gene Expression Omnibus database (GEO, https://www.ncbi.nlm.nih.gov/geo/), which contained 12 chemotherapy-resistant and 16 chemotherapy-sensitive high-grade serous ovarian carcinoma samples [53].

### Xenograft mouse model

The study on animal subjects was approved by the Ethics Committee of Shanghai Public Health Clinical Center. A total of 5 × 10^6^ cells in 100 μl medium were subcutaneously injected into 5-week-old female BALB/c nude mice (*n* = 5/group, Shanghai Super-B&K Laboratory Animal Corp. Ltd., Shanghai, China). PTX (20 mg/kg) was administered by inter-tumor injection every 3 days for 3 weeks when the tumor reached a volume of approximately 80–100 mm^3^. Tumor volume was calculated as described previously [50]. Tumor size was measured using the vernier caliper. For tumor metastasis assay, 1.5 × 10^6^ cells per mice were injected intraperitoneally into 5-week old female nude mice (*n* = 5/group). PTX (15 mg/kg) was administered by intraperitoneal injection every 3 days for 3 weeks. Metastasis nodes and organs were collected.

### Statistical analysis

Statistical analyses were performed with GraphPad Prism 8. Statistical significance was determined using an unpaired two-tailed Student’s *t*-test or one-way ANOVA, followed by the Tukey test according to the type of experiment. A χ^2^-test was applied to analyze the relationship between SRI and ZEB1. Statistical significance was considered when *P* < 0.05.

## Supplementary information

Supplementary Methods

Supplementary Figures

Supplementary Tables
