# Supplementary material for: A novel homeostatic loop of sorcin drives paclitaxel-resistance and malignant progression via Smad4/ZEB1/miR-142-5p in human ovarian cancer
Source: Oncogene. 2021 Jun 23;40(30):4906–18. doi: 10.1038/s41388-021-01891-6 (PMC8321900; doi:10.1038/s41388-021-01891-6)
Supplement: Supplementary file 1 — Supplementary Methods [file 41388_2021_1891_MOESM1_ESM.pdf]

Supplementary Methods

Microarray and proteomics

Total RNAs from OVCAR-3 and OV3R-PTX cells were extracted using TRIzol reagent (Invitrogen). RNA samples were randomly assigned numbers and carried out by CapitalBio Technology (Beijing, China). Agilent human gene expression microarray V4.0 was performed according to the manufacturer's instructions. The differentially expressed genes were screened with threshold criteria of adjusted $P < 0.05$ and fold change ≥ 2. For proteomics analysis, cell lysates in RIPA lysis buffer were applied for mass spectrometric detection carried out at Fudan University.

Transfection of siRNA, miRNA, and plasmid and infection of shRNA lentivirus

Small interfering RNAs (siRNAs) for human SRI and ZEB1 and corresponding negative controls (NC) were synthesized in GenePharma (Shanghai, China). The miR-142-5p and miR-147a mimics, inhibitors, and corresponding controls (Ctrl) were purchased from GenePharma. SRI and ZEB1 overexpressing plasmids were constructed in mammalian expression vectors pcDNA4.1/TO/myc-His (B) and pcDNA3.1 (Invitrogen), respectively. The cells were transfected with siRNAs, mimics, inhibitors, and plasmids or their NCs using X-tremeGENE Transfection Reagent (Roche Diagnostics, Indianapolis, IN, USA) or Lipo8000™ Transfection Reagent (Beyotime) following the manufacturer's protocols. SRI short hairpin RNA (shRNA) was cloned into pHY-310 RNAi lentivirus (Hanyin Biotechnology Co., Ltd., Shanghai, China).

Quantitative RT-PCR

Total RNA was extracted from cultured cells using the RNA-Quick Purification Kit (Yishan Biotechnology Co., Ltd., Shanghai, China) according to the manufacturer's instructions. Total mRNA, primary miRNA, and mature miRNA were reversely transcribed and PCR was performed as described previously.

Extraction of the total, cytoplasmic, and nuclear proteins and western blot

Total protein was extracted with SDS lysis buffer (Thermo Fisher Scientific, Inc.) supplemented with the proteinase inhibitors (Beyotime) and phosphatase inhibitor (Nanjing KeyGen Biotech Co., Ltd.). The cytoplasmic and nuclear proteins were extracted with an extraction kit (#SC-003; Invent Biotechnologies, Inc) according to the manufacturer's instructions. Western blot analysis was performed as described previously. Immunoblots were quantified using Tanon-4500 Gel Imaging System with GIS ID Analysis Software v4.1.5 (Tanon Science & Technology Co., Ltd., Shanghai, China).

Immunohistochemical staining (IHC)

Briefly, the 4% paraformaldehyde-fixed, paraffin-embedded tissue specimens were sectioned (4 μm thick), deparaffinized in xylene, and rehydrated in a descending alcohol series. Endogenous peroxide activity was quenched by 3% hydrogen peroxide in methanol for 15 min. After blocking with 10% normal goat serum for 40 min at room temperature, the section was incubated with an anti-SRI antibody (1:300 dilution, Abcam) at 4°C overnight, followed by incubation with biotinylated anti-rabbit secondary antibody at room temperature for 1 h. After washing, the signal was detected using a DAB kit. The immunoreactive staining of SRI was evaluated by the scores of

the sum of the percentage of positive cells (0, <5%; 1, 5-25%; 2, 25-50%; 3, 50-75%; and 4, >75%) and the intensity score (0, no coloration; 1, pale brown; 2, brown; and 3, dark brown). The final scores of more than 2 were considered positive.

Immunofluorescence (IF) and confocal microscopy

After plating cells onto coverslips in a 24-well plate overnight, cells were fixed with 4% paraformaldehyde (PFA) for 30 min and permeabilized with 0.1% Triton X-100 in PBS for 15 min. After blocking with goat serum, cells were incubated with primary antibodies dilution in 3% BSA at 4°C overnight, followed by secondary antibody incubation for 1 h at room temperature in the dark. DAPI (Beyotime) was added for nuclei staining. Images were captured by a fluorescence microscope (Olympus Corporation, Tokyo, Japan). For confocal microscopy assays, cells were grown in confocal dishes (Beyotime) overnight. Following the above procedures, images were captured by a confocal laser-scanning microscope (Leica SP5, Wetzlar, Germany).

Flow cytometry

The apoptotic cells were detected by Annexin V conjugated with fluorescein isothiocyanate (BD Pharmingen) and propidium iodide (PI) complied with the manufacturer's instructions and counted by flow cytometry (Beckman Coulter, Inc.). For cell cycle-detecting, cells were fixed in 70% ice-cold ethanol in PBS at 4˚C overnight. After cells were resuspended in 500 μl of PI/RNase Staining Buffer (BD Pharmingen), 2×10^4 cells were analyzed by flow cytometry with ModFit LT software v4.1.7 (Verity Software House, Inc.).

Paclitaxel cytotoxicity assay

Cells were seeded at a 96-well plate at a density of 1×10^4 cells/well and then treated with different doses of PTX for 48 h. Cell growth was detected using a CCK-8 kit. The relative cell viability (%) was calculated as a percentage of viable cell proportion for PTX-treated cells *vs*. untreated control cells.

Spheroid formation assay

OVCAR-3 (1×10^3) and SK-OV-3 (3×10^3) cells were seeded in 6-well ultra-low attachment plates (Corning Incorporated, Corning, NY, USA) containing serum-free medium DMEM/F12 (Gibco-Invitrogen) supplemented with B27 (1:50, ThermoFisher), heparin (1:100, Sigma-Aldrich), EGF (10 ng/ml, ThermoFisher), and bFGF (20 ng/ml, ThermoFisher) as described previously. The images were captured after 2 weeks of cell culture. The size of spheroids was evaluated by measuring two orthogonal diameters (d1 and d2) using CellSens Life Science Imaging Software (CellSens Dimension 1.12; Olympus Corporation, Tokyo, Japan). The mean diameter of a spheroid was calculated by the formula of √d1d2.

Dual-luciferase reporter assay

The HEK 293T cells were co-transfected with wild-type (WT) or mutated (Mut) SRI 3'-UTR pMIR-report vectors (GeneCopoeia, Inc.), miR-142-5p mimics, miR-142-5p inhibitors, or their negative controls for 24 h. The luciferase activities were measured with the Luc-Pair™ Duo-Luciferase Assay Kit (GeneCopoeia, Inc.). For the miR-142 promoter deletion assay, four different pieces of miR-142 promoter regions containing different ZEB1 binding sites were amplified and ligated into the pGL4-Basic vector

(Promega, USA). pGL4-miR-142, NC-siRNA, or ZEB1-siRNA, and the control Renilla luciferase vector pRL-TK were cotransfected into SK3R-PTX and OV3R-PTX cells. The luciferase activity was measured after 48 h transfection.
