# Supplementary material for: A novel homeostatic loop of sorcin drives paclitaxel-resistance and malignant progression via Smad4/ZEB1/miR-142-5p in human ovarian cancer
Source: Oncogene. 2021 Jun 23;40(30):4906–18. doi: 10.1038/s41388-021-01891-6 (PMC8321900; doi:10.1038/s41388-021-01891-6)
Supplement: Supplementary file 2 — Supplementary Figures [file 41388_2021_1891_MOESM2_ESM.pdf]

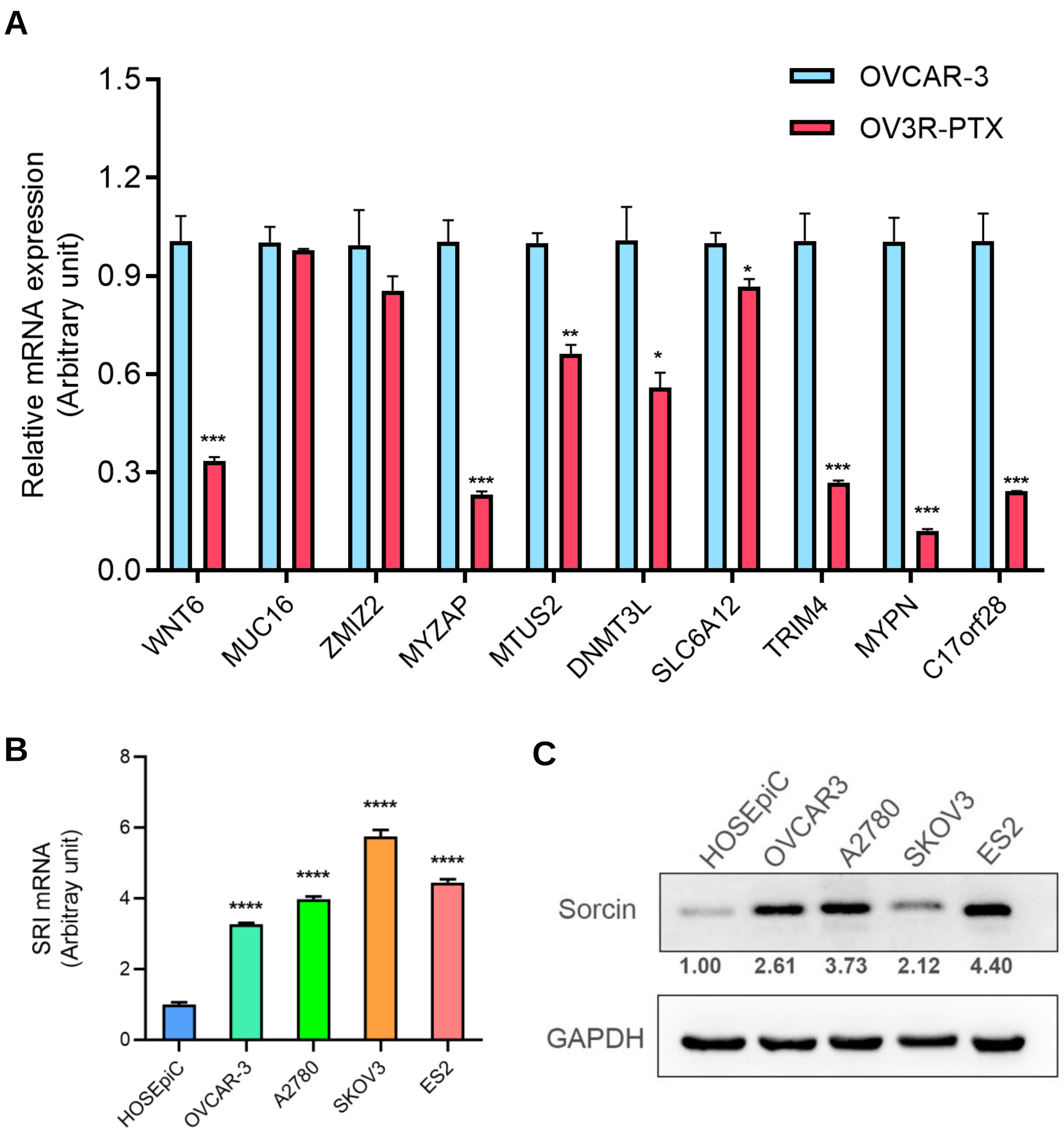

Supplementary Figure S1.
Detection of top 10 downregulated mRNAs in PTX-resistant cells and SRI expression in human ovarian cells. **A**, Top 10 downregulated mRNAs in OV3R-PTX cells compared with OVCAR-3 cells detected by microarray were vilified by qRT-PCR. **B**, SRI mRNA in normal ovarian surface epithelial cells (HOSEpiC) *vs*. four ovarian cancer cells by qRT-PCT. **C**, SRI protein expression in HOSEpiC *vs*. four ovarian cancer cells by Western blot.

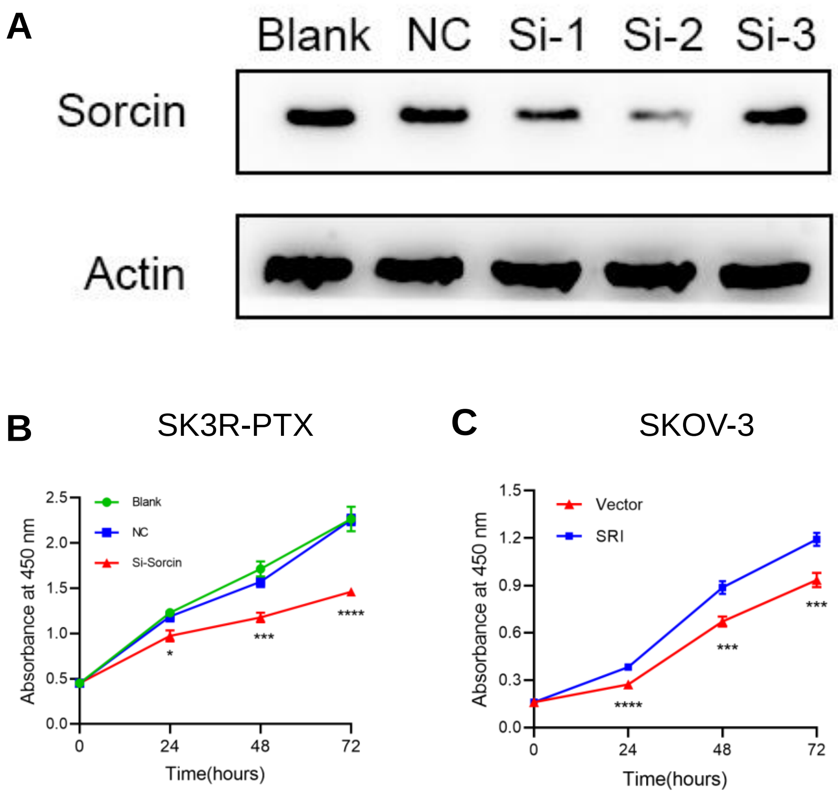

Supplementary Figure S2.

Effect of SRI on cell proliferation. **A**, Examination of the efficacy of SRI-siRNA in OV3R-PTX cells by Western blot. three SRI-specific siRNAs (Si-1 to -3) were designed to silence SRI expression. **B**, Cell proliferation of SK3R-PTX after sorcin-siRNA (Si-2) transfection detected by the CCK8 assay. **C**, SKOV-3 cell proliferation after sorcin plasmid transfection detected by the CCK8 assay.

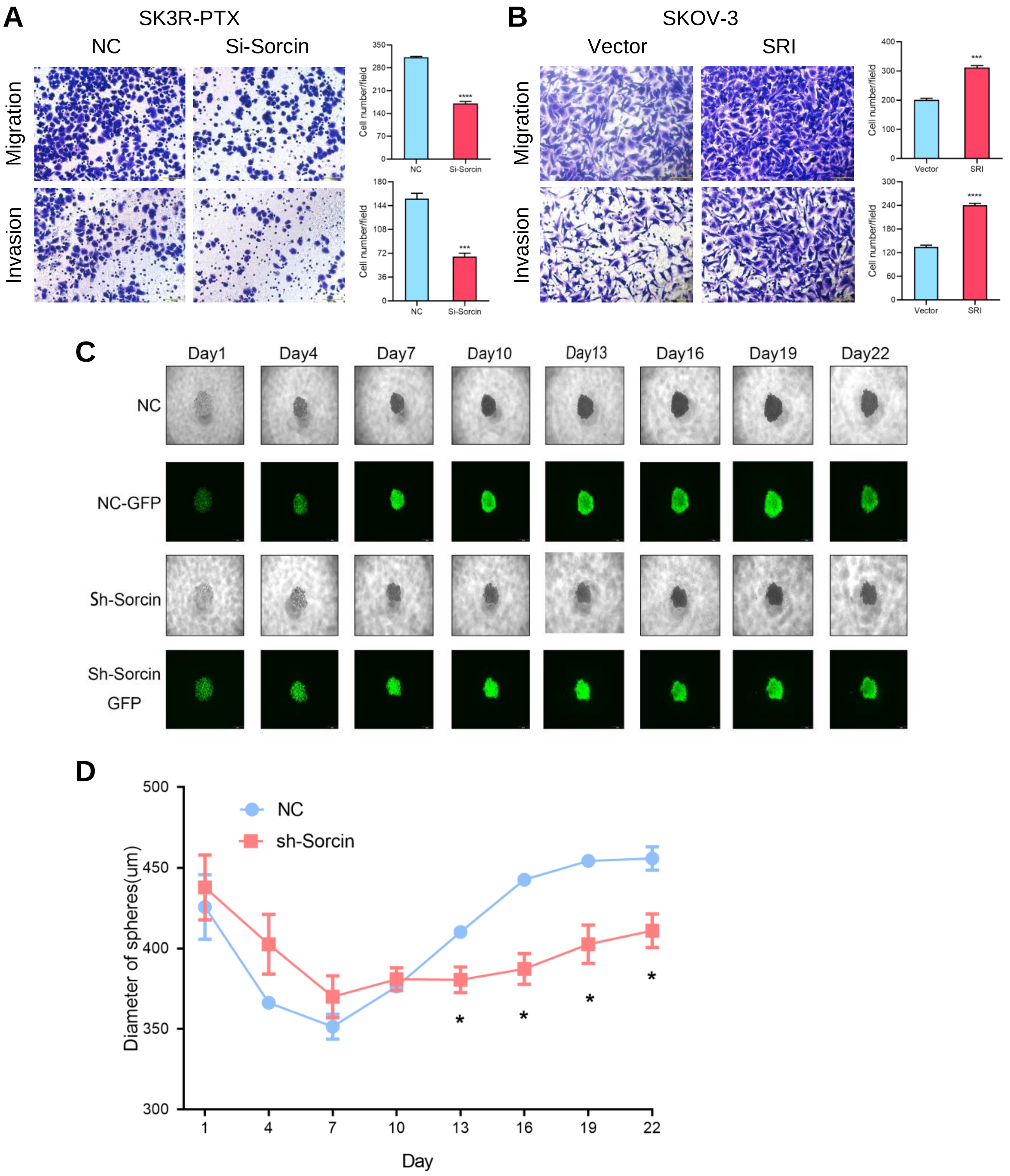

Supplementary Figure S3

Effect of SRI on cell migration, invasion, and Tumor spheroid formation. **A**, Migration and invasion of SK3R-PTX cells after the knockdown of sorcin by siRNA for 48 h. **B**, Migration and invasion of SKOV-3 cell after the transfection of sorcin plasmid for 48 h. **C**, Tumor spheroid formation using a three-dimensional cell culture system after SRI-shRNA infection in OV3R-PTX cells. **D**, Measurement of sphere size. The knockdown of SRI decreased the size of a tumor spheroid in OV3R-PTX cells. Scale bar, 100 μm.

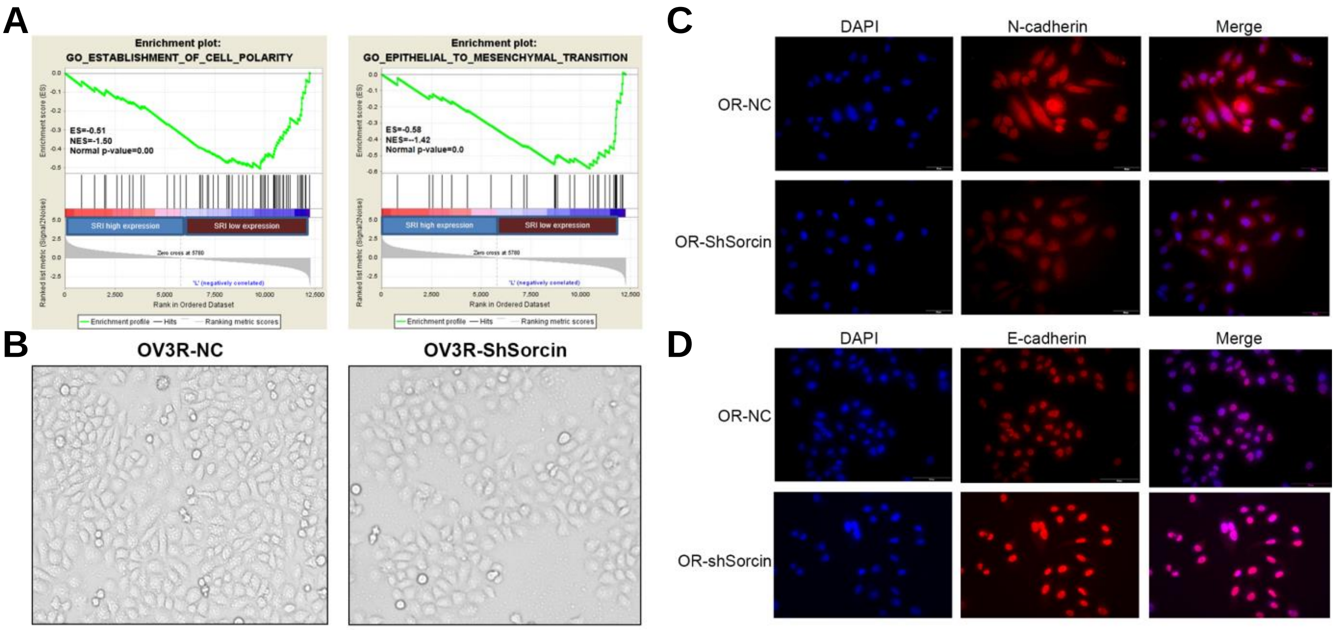

Supplementary Figure S4.

Detection of epithelial-mesenchymal transition (EMT) in OV3R-PTX cells. **A**, GSEA analyses. GSEA revealed that Sorcin (SRI) participated in the establishment of cell polarity (left) and EMT (right). **B**, Phase-contrast imaging showed the morphology of control cells (OV3R-NC) and SRI-knockdown cells (OV3R-ShSorcin). **C**, Comparison of N-cadherin expression between OV3R-ShSorcin cells and negative control cells detected by immunofluorescence staining. **D**, Comparison of E-cadherin expression between OV3R-ShSorcin cells and negative control cells detected by immunofluorescence staining.

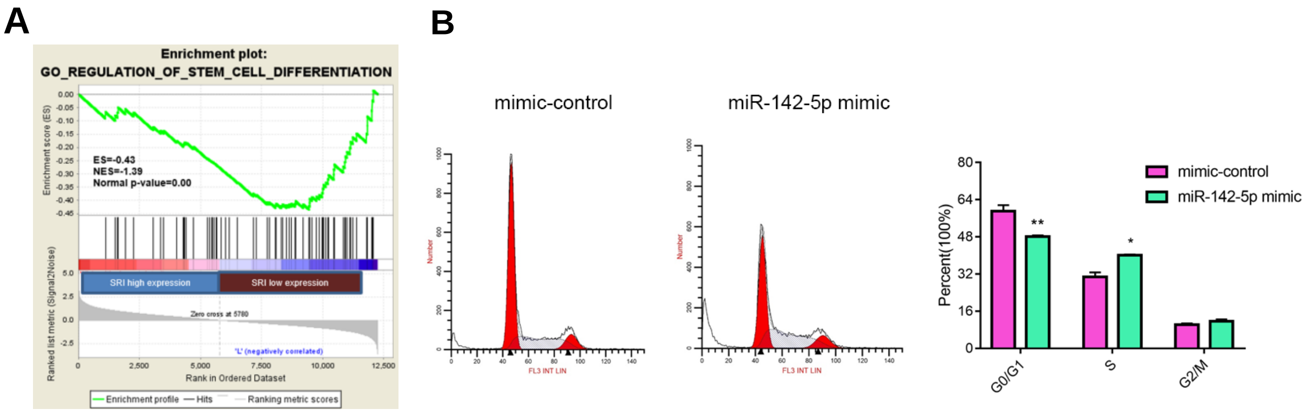

Supplementary Figure S5.

Gene Set Enrichment Analysis (GSEA) and flow cytometry analysis of cell cycle. **A**, Gene Set Enrichment Analysis of significant enrichment in the regulation of stem cell differentiation with different expression of SRI. **B**, Flow cytometry analysis of cell cycle. SK3R-PTX cells were transfected with miR-142-5p mimics or its control for 2 days. The cell cycle was determined by flow cytometry.

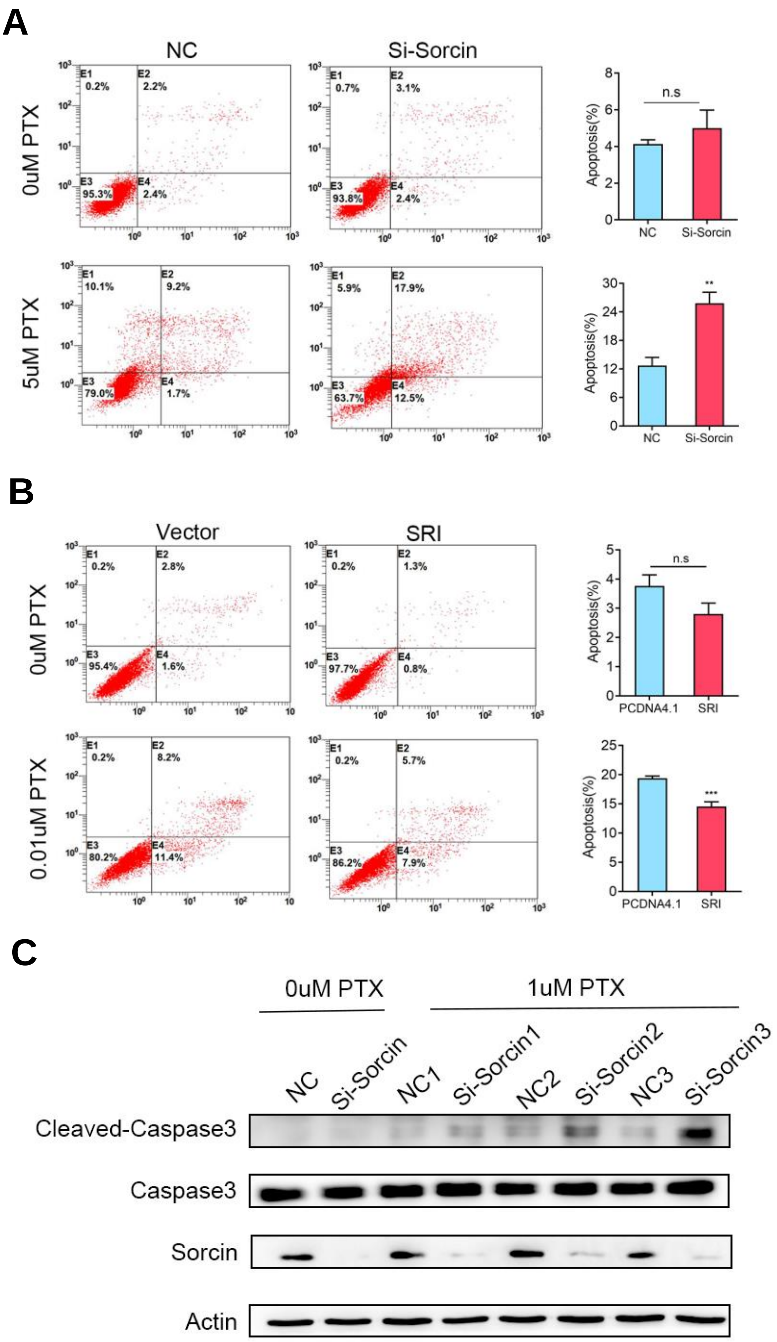

Supplementary Figure S6.
Detection of apoptotic cells. **A**, OV3R-PTX cells were transfected with Sorcin-siRNA or negative control-siRNA (NC) in the presence or absence of 5 μM paclitaxel (PTX). Apoptotic cells were detected by flow cytometry. **B**, OVCAR-3 cells were transfected with Sorcin-overexpressing plasmid (SRI) or control vector in the presence or absence of 0.01 μM PTX. Apoptotic cells were detected by flow cytometry. n.s., not significant difference between two groups. **, $P < 0.01$; ***, $P < 0.001$. **C**, Detection of apoptotic protein by Western blot. OV3R-PTX cells were transfected with SRI-siRNA or negative control (NC) in the presence or absence of paclitaxel (PTX).

A

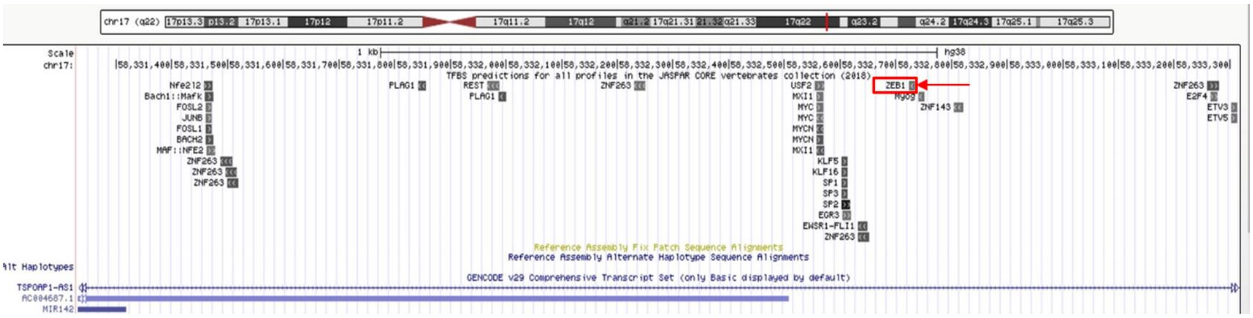

B

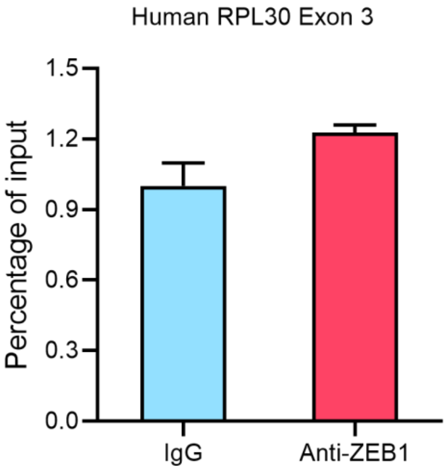

Supplementary Figure S7.

Prediction of transcription factors and ChIP-qPCR assay for negative control. **A**, Prediction of transcription factors for miR-142-5p expression. The UCSC Genome Browser database combining with the JASPAR database were applied to identify transcription factors that bind to the miR-142-5p promoter region (2 kb upstream of the transcriptional start site). Several transcription factors are shown, including ZEB1 as indicated in red rectangular frame. **B**, Negative control of ChIP-qPCR assay. ZEB1 did not bind to the RPL30 Exon 3 in SK3R-PTX cells.

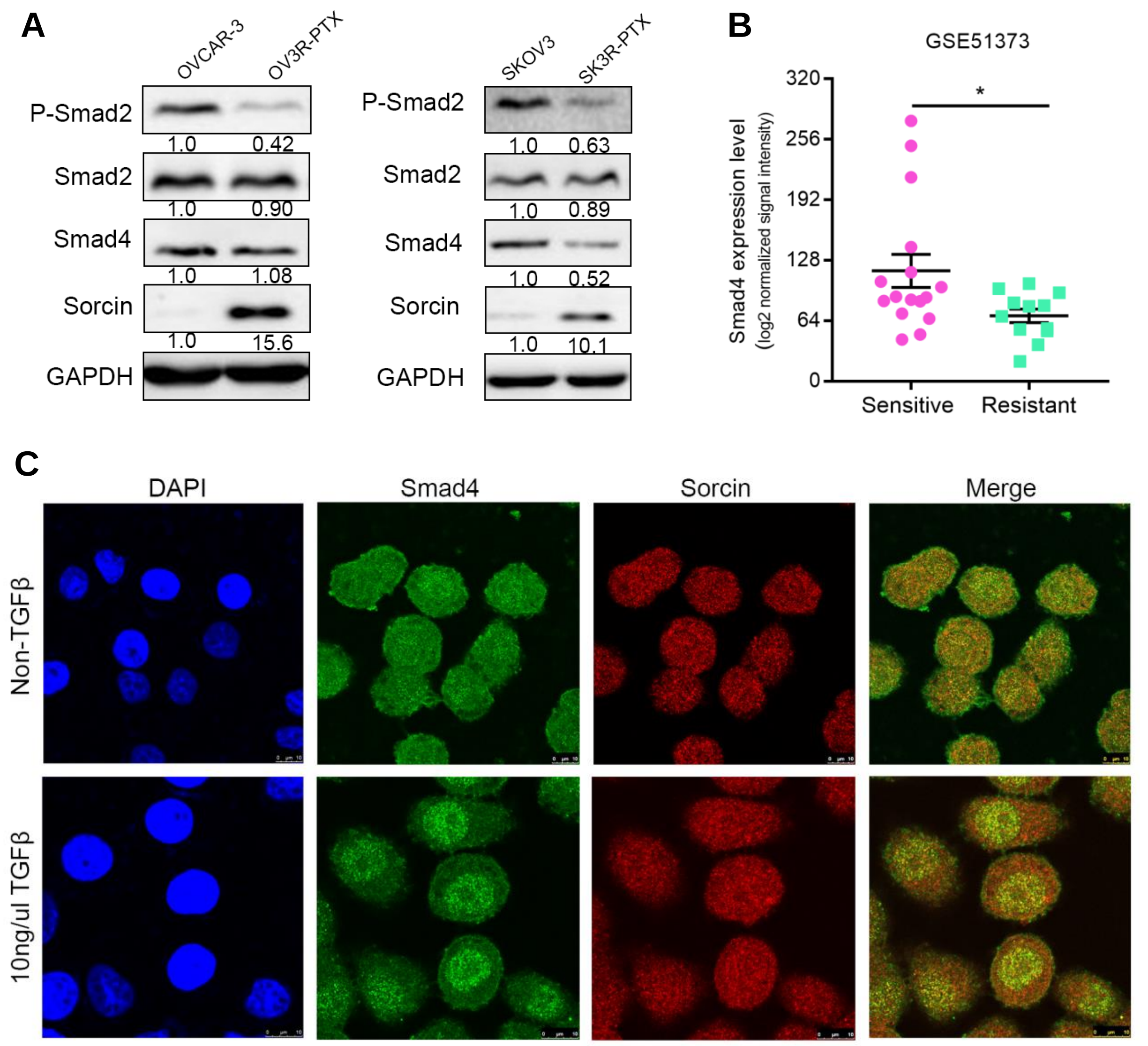

Supplementary Figure S8.

Expression of Smad in chemo-sensitive and chemo-resistant ovarian cancer. **A**, Detection of specific proteins in ovarian cancer cells (OVCAR-3 and SKOV3) and PTX-resistant cells (OV3R-PTX and SK3R-PTX) by Western blot. **B**, Expression of Smad4 in chemo-resistant and sensitive ovarian cancers. Data were extracted from the GSE51373 dataset. **C**, Detection of Smad4 and Sorcin co-localization in SK3R-PTX cells in the presence or absence of TGF-β1 by confocal microscopy. Alexa Fluor 488 (green) or Alexa Fluor 594 (red) was used to detect Smad4 and Sorcin, respectively. The images of Smad4 and Sorcin were merged. The nucleus was stained with DAPI.
