# Supplementary material for: A novel homeostatic loop of sorcin drives paclitaxel-resistance and malignant progression via Smad4/ZEB1/miR-142-5p in human ovarian cancer
Source: Oncogene. 2021 Jun 23;40(30):4906–18. doi: 10.1038/s41388-021-01891-6 (PMC8321900; doi:10.1038/s41388-021-01891-6)
Supplement: Supplementary file 3 — Supplementary Tables [file 41388_2021_1891_MOESM3_ESM.pdf]

Supplementary Table S1. The top 50 upregulated proteins detected by mass spectrometry in OV3R-PTX cells compared to OVCAR-3 cells

Protein	Full name	Accession Number
KDM6A_HUMAN	Lysine-specific demethylase 6A	O15550
MDR1_HUMAN	ATP-dependent translocase ABCB1	P08183
SORCN_HUMAN	Sorcin	P30626
TTYH3_HUMAN	Protein tweety homolog 3	Q9C0H2
TMM97_HUMAN	Sigma intracellular receptor 2	Q5BJF2
PRP18_HUMAN	Pre-mRNA-splicing factor 18	Q99633
CNOT8_HUMAN	CCR4-NOT transcription complex subunit 8	Q9UFF9
FKBP5_HUMAN	Peptidyl-prolyl cis-trans isomerase FKBP5	Q13451
AK1C2_HUMAN	Aldo-keto reductase family 1 member C2	P52895
PHP14_HUMAN	14 kDa phosphohistidine phosphatase	Q9NRX4
PURA1_HUMAN	Adenylosuccinate synthetase isozyme 1	Q8N142
AN30B_HUMAN	Ankyrin repeat domain-containing protein 30B	Q9BXX2
MTCH2_HUMAN	Mitochondrial carrier homolog 2	Q9Y6C9
UB2E1_HUMAN	Ubiquitin-conjugating enzyme E2 E1	P51965
EF1A2_HUMAN	Elongation factor 1-alpha 2	Q05639
NJMU_HUMAN	Protein Njmu-R1	Q9HAS0
IDE_HUMAN	Insulin-degrading enzyme	P14735
MRGBP_HUMAN	MRG/MORF4L-binding protein	Q9NV56
H2B1O_HUMAN	Histone H2B type 1-O	P23527
VP33A_HUMAN	Vacuolar protein sorting-associated protein 33A	Q96AX1
NFKB2_HUMAN	Nuclear factor NF-kappa-B p100 subunit	Q00653
PTPM1_HUMAN	Phosphatidylglycerophosphatase and protein-tyrosine phosphatase 1	Q8WUK0
PARG_HUMAN	Poly(ADP-ribose) glycohydrolase	Q86W56
H2B1H_HUMAN	Histone H2B type 1-H	Q93079
CT451_HUMAN	Cancer/testis antigen family 45 member A1	Q5HYN5
ABCBB_HUMAN	Bile salt export pump	O95342
QSER1_HUMAN	Glutamine and serine-rich protein 1	Q2KHR3
PA24A_HUMAN	Cytosolic phospholipase A2	P47712
TPP1_HUMAN	Tripeptidyl-peptidase 1	O14773
SRSF3_HUMAN	Serine/arginine-rich splicing factor 3	P84103
GPX1_HUMAN	Glutathione peroxidase 1	P07203
PTN23_HUMAN	Tyrosine-protein phosphatase non-receptor type 23	Q9H3S7
S10AB_HUMAN	Protein S100-A11	P31949

AGAP3_HUMAN	ANK repeat and PH domain-containing protein 3	Q96P47
LYAR_HUMAN	Cell growth-regulating nucleolar protein	Q9NX58
TBB5_HUMAN	Tubulin beta chain	P07437
CK083_HUMAN	Ubiquinol-cytochrome-c reductase complex assembly factor 3	Q6UW78
SIN3B_HUMAN	Paired amphipathic helix protein Sin3b	O75182
NDRG1_HUMAN	Protein NDRG1	Q92597
MLEC_HUMAN	Malectin	Q14165
SF3B5_HUMAN	Splicing factor 3B subunit 5	Q9BWJ5
GSTM3_HUMAN	Glutathione S-transferase Mu 3	P21266
PRRC1_HUMAN	Protein PRRC1	Q96M27
GLP3L_HUMAN	Golgi phosphoprotein 3-like	Q9H4A5
LEG1_HUMAN	Galectin-1	P09382
CSTF2_HUMAN	Cleavage stimulation factor subunit 2	P33240
CYB5_HUMAN	Cytochrome b5	P00167
HPBP1_HUMAN	Hsp70-binding protein 1	Q9NZL4
STAT6_HUMAN	Signal transducer and activator of transcription 6	P42226
GBB4_HUMAN	Guanine nucleotide-binding protein subunit beta-4	Q9HAV0

Supplementary Table S2. Sequences of siRNA, shRNA, and oligonucleotides

Name	Sequence (5' → 3')	Target position
SRI-siRNA		
Sense-1	GCAUUGCUGGAGGAUACAATT	nt 197-215
Antisense-1	UUGUAUCCUCCAGCAAUGCTT	
Sense-2	GCUGGAGACAACACUUUAUTT	nt 323-341
Antisense-2	AUAAAGUAUUAUCUCCAGCTT	
Sense-3	CCUGACAACAAUGGGAUUUTT	nt 396-414
Antisense-3	AAAUCCCAUUGUUGUCAGGTT	
NC-siRNA		
Sense	UUCUCCGAACGUGUCACGUTT	Scramble
Antisense	ACGUGACACGUUCGGAGAATT	
ZEB1-siRNA		
Sense	GCUGAGAAGCCUGAGUCCUTT	nt 1715-1733
Antisense	AGGACUCAGGCUUCUCAGCTT	
SMAD4-siRNA		
Sense	GCUCCUAGACGAAGUACUUTT	nt 2140-2158
Antisense	AAGUACUUCGUCUAGGAGCTT	
SRI-shRNA		
Sense	gatccGCTGGAGACAACACTTTAT TTCAAGAGAATAAAGTGTTGTCT CCAGCTTTTTTg	
Antisense	aattcAAAAAAGCTGGAGACAACA CTTTATTCTCTTGAAATAAAGTG TTGTCTCCAGCg	
miRNA		
miR-Ctrl	UUCUCCGAACGUGUCACGUTT	Scramble
miR-142-5p mimic	CAUAAAGUAGAAAGCACUACU	
miR-147a mimic	GUGUGUGGAAAUGCUUCUGC	
miR-142-5p inhibitor	AGUAGUGCUUUCUACUUUAUG	

Ctrl, control; NC, negative control; nt, nucleotide; shRNA, short hairpin RNA; siRNA, small interfering RNA. The target position in the SRI mRNA (GenBank Accession: NM_003130), ZEB1 mRNA (GenBank Accession: NM_030751.6), and SMAD4 mRNA (GenBank Accession: NM_005359.6) sequences are shown.

Supplementary Table S3. Sequences of RT-PCR primers

Primer name	Sequence (5’ → 3’)	Target position
SRI		
Forward	GGACAAACTCAGGATCCGCTGTA	nt 97-119
Reverse	GCCGGCAAGTCTCCAGGTTA	nt 222-241
ZEB1		
Forward	GATGATGAATGCGAGTCAGATGC	nt 344-366
Reverse	ACAGCAGTGTCTTGTTGTTGT	nt 409-429
GAPDH		
Forward	GCACCGTCAAGGCTGAGAAC	nt 249-268
Reverse	TGGTGAAGACGCCAGTGGA	nt 386-368
RT primer		
miR-142-5p	GTCGTATCCAGTGCAGGGTCCGAG GTATTCGCACTGGATACGACagtagt	
miR-147a	GTCGTATCCAGTGCAGGGTCCGAG GTATTCGCACTGGATACGACgcagaa	
Pri-miR-142		
Forward	TGGAGCAGGAGTCAGGAGG	nt 949-967
Reverse	GCCGAGGAAGATGGTGG	nt 1132-1148
U6		
Forward	CTCGCTTCGGCAGCACA	
Reverse	AACGCTTCACGAATTTGCGT	
miR142-Chip-EBOX1		
Forward	CCTTCCCTCGAGCCTCTTTC	
Reverse	GGCTAGGTGTTCACGCTCTC	
miR142-Chip-EBOX2		
Forward	CTGAGTCAGTCCCAGGCTCTTG	
Reverse	GGGAGGACAGACTGACAGGAAAC	
miR142-Chip-ZBOX		
Forward	GTGGCCTCGGGGTTATAAGC	
Reverse	CAGTTGGGGCAGCAGTGAAG	

The positions in SRI mRNA (GenBank Accession: NM_003130), ZEB1 mRNA (GenBank Accession: NM_030751.6), GAPDH mRNA (GenBank Accession: NM_001256799), and Pri-miR-142 (GenBank Accession: LC194526.1) sequences are shown. nt, nucleotide.

Supplementary Table S4. List of antibodies

Antibody	Source	Dilution	Company (Catalog #)	Application
SRI	Rabbit pAb	1:300-2000	abcam (ab71983)	WB, IF, IHC
SRI	Rabbit pAb	3 μg	Proteintech (16471-1-AP)	IP
Claudin-1	Rabbit mAb	1:1000	CST (13255S)	WB
N-cadherin	Rabbit pAb	1:200-1000	Immunoway (YT2988)	WB, IF
E-cadherin	Mouse mAb	1:200-1000	CST (14472S)	WB, IF
ZO-1	Rabbit pAb	1:1000	Proteintech (21773-1-AP)	WB
PARP	Rabbit mAb	1:3000	CST (9532S)	WB
Caspase3	Rabbit IgG	1:1000	CST (9662)	WB
ZEB1	Rabbit mAb	1:1000	CST (3396)	WB, Chip
Smad2	Rabbit mAb	1:2000	CST (5339S)	WB
P-Smad2	Rabbit mAb	1:2000	CST (3108)	WB
Smad4	Rabbit mAb	1:1000	CST (46535)	WB, IF
Smad4	Mouse mAb	2 μg	Santa Cruz (sc-7966)	IP
CD44	Mouse mAb	1:500-5000	CST (3570)	WB, IF
CD133	Rabbit mAb	1:1000	CST (64326)	WB
SOX2	Rabbit mAb	1:1000	CST (3579)	WB
Cyclin B1	Mouse mAb	1:2000	CST (4135S)	WB
Cyclin D1	Rabbit mAb	1:1000	CST (2978S)	WB
Cyclin E1	Mouse mAb	1:1000	CST (4129)	WB
GAPDH	Mouse mAb	1:5000	Proteintech (60004-1-IG)	WB
β-actin	Mouse mAb	1:5000	Proteintech (66009-1-IG)	WB
Lamin B1	Rabbit pAb	1:2000	Proteintech (12987-1-AP)	WB
Alexa-Fluor 488	Mouse IgG	1:200	CST (4408)	IF
Alexa-Fluor 594	Rabbit IgG	1:200	CST (8889)	IF

Chip, Chromatin immunoprecipitation; CST, Cell Signaling Technology, Inc.; IF, Immunofluorescence; IHC, Immunohistochemical staining; IP, Immunoprecipitation; mAb, monoclonal antibody; pAb, polyclonal antibody; WB, Western blot.
